# Fabrication techniques and biomedical applications of strontium-based nanofibers

**DOI:** 10.1186/s12951-026-04112-y

**Published:** 2026-03-24

**Authors:** Esraa Dakrory Mahmoud, Hoda Elkhenany, Tarek M. Bedair

**Affiliations:** 1 Chemistry Department, Faculty of Science, Minia University, El-Minia, 61519 Egypt; 2 Department of Surgery, Faculty of Veterinary Medicine, Alexandria University, Qetaa an Nahdah, Moharam Bek, Alexandria, 5410012 Governorate Egypt

**Keywords:** Strontium, Nanofibers, Electrospinning, Biomaterials, Bone regeneration, Tissue engineering

## Abstract

Strontium (Sr)-based nanofibers have gained great attention in biomedical and tissue engineering applications due to their unique ability to combine nanoscale structural features with the biological activity of Sr ions (Sr^2+^). Nanofibers offer a versatile platform to harness these properties owing to their high surface area, tunable porosity, and mechanical strength. The incorporation of Sr^2+^ ions further enhances their bio-functionality and offers a cost-effective alternative to growth factor-based strategies. Sr^2+^ ions could stimulate the production of growth factors such as vascular endothelial growth factor (VEGF) and basic fibroblast growth factor (bFGF), thereby promoting neovascularization, while also enhancing osteogenesis by mimicking calcium’s physiological role, inducing mesenchymal stem cell differentiation, and stimulating extracellular matrix mineralization. This review summarizes recent advances in the fabrication techniques such as electrospinning, assisted-electrospinning, and non-electrospinning, including the design, control composition, morphology, and functionality of Sr-based nanofibers. The mechanisms governing Sr^2+^ ions interactions with cells and tissues are discussed, along with in vitro and in vivo biological outcomes. Our bibliometric analysis shows that Sr-based nanofibers have been most extensively investigated in bone tissue engineering, followed by applications in drug delivery and tumor therapy, with fewer studies exploring skin and cartilage regeneration. This review highlights the advantages and disadvantages of every fabrication strategy, discusses biomedical applications of Sr-based nanofibers, and outlines challenges and future directions for their clinical translation.

**Graphical Abstract:**

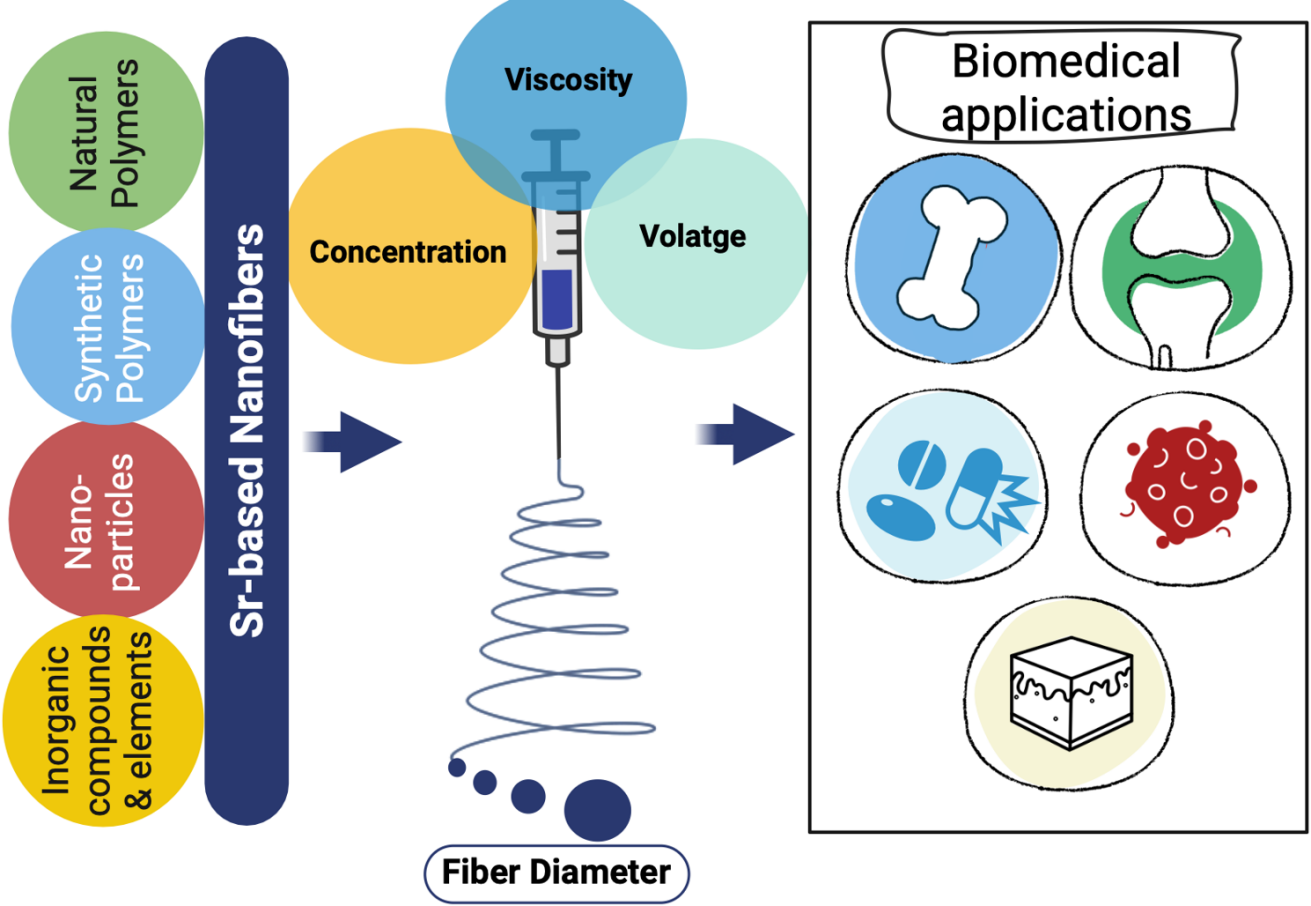

## Introduction

Nowadays, nanotechnology and nanoscience are receiving a lot of interest due to their multiple applications in various fields such as agriculture and medicine [1, 2]. Nanoscience is considered as the study of materials at nanoscale level; atomic and molecular state [3]. Nanomaterials are divided into zero dimensional, one dimensional, two dimensional and three-dimensional nanomaterials [4, 5]. While, nanotechnology is the field which deals with production, design and use of nanomaterials and related devices [6]. Nanotechnology is focusing on benefit the advantages of noble properties of the materials at nanoscale for innovative applications [7]. It has an efficient role in various fields such as biomedicine [8] as it can be used in the diagnosis, the therapy of many diseases, and the production of many medical related materials [9]. Furthermore, it has been used to improve environments, such as the production of efficient energy with low pollutants, the fabrication of solar cells to generate electricity, and the removal of organic contaminants from groundwater and air [10]. Moreover, designed polymeric nanobrushes could enhance the interfacial interaction between the polymer coating and metallic surface which enhance the efficacy of drug eluting stents [11, 12].

Nanoparticles (NPs) have attracted huge amount of interest due to high surface to volume ratio, reactivity, sensitivity, and the ability to modify their surfaces. However, they have some disadvantages that limit their applicability such as high chemical activity and toxicity owing to the greater surface area and small size that enables their cell uptake, interactions with biomolecules, and tissues [13, 14]. In addition, the bioaccumulation of NPs in the surrounding tissue could increase the release of toxic ions that could generate reactive oxygen species (ROS) which subsequently destroy the DNA and increase the risk of cancer [14]. In contrast, nanofibers show a synergistic effect by either loading NPs within them [15], or decorating NPs on the surface of nanofibers [16] and releasing the NPs in controlled manner with minimal dosage that reduce their toxicity.

This review article shed light on nanofibers as a one-dimensional nanomaterial due to their significant effect and unique features. Nanofibers have large surface area-to-volume ratio, available to be synthesized at room temperature, high porosity, strong mechanical properties, gas and nutrient permeability, and releasing bioactive materials that support cell attachment, spreading, proliferation, migration, and infiltration [17, 18]. Additionally, electrospun nanofiber scaffolds have an internal porous network structure make them mimic the extracellular matrix (ECM) and human tissue structures, which supports cell growth [19–21]. Many polymers and substances such as natural polymers, synthetic polymers, carbon-based nanomaterials, semiconductor nanomaterials and composite nanomaterials can be used for the fabrication of nanofibers [7, 22, 23]. Altogether, this makes them promising candidate for various advanced applications such as water treatment, energy production and storage, solar systems, tissue engineering, drug delivery, wound dressing, etc [24–28]. This broad spectrum of applications and unique features of nanofibers highlights the growing interest and continuous innovations within the field of nanofibrous materials.

In recent years, we found that the incorporation of inorganic compounds with angiogenic, osteogenic and therapeutic effect into biomaterial has become more and more popular compared to growth factor due to their low cost, high stability and clinically safe [29, 30]. Many inorganic ions such as magnesium (Mg^2+^), calcium (Ca^2+^), and strontium (Sr^2+^) play an important role in cell fate and physiological behavior such as angiogenesis and/or osteogenesis [31]. Among inorganic ions, Sr^2+^ ions have proved the potential effect to stimulate proangiogenic factors such as vascular endothelial growth factor (VEGF), basic fibroblast growth factor (bFGF) and matrix metalloproteinase-2 [30]. Furthermore, Sr^2+^ ions could promote the proliferation and migration of endothelial cells which promotes the formation of tubular structure and consequently enhances angiogenesis [32, 33]. In the term of osteogenesis, Sr^2+^ ions is very similar to Ca^2+^ ions which enables Sr^2+^ ions to replace Ca^2+^ ions in some physiological functions such as muscles contraction, blood clotting, and release of certain hormone [31, 34]. Additionally, Sr^2+^ ions could induce mesenchymal stem cell proliferation and osteogenic differentiation, and increases the ECM)deposition and mineralization [31, 35, 36].

**Fig. 1:**
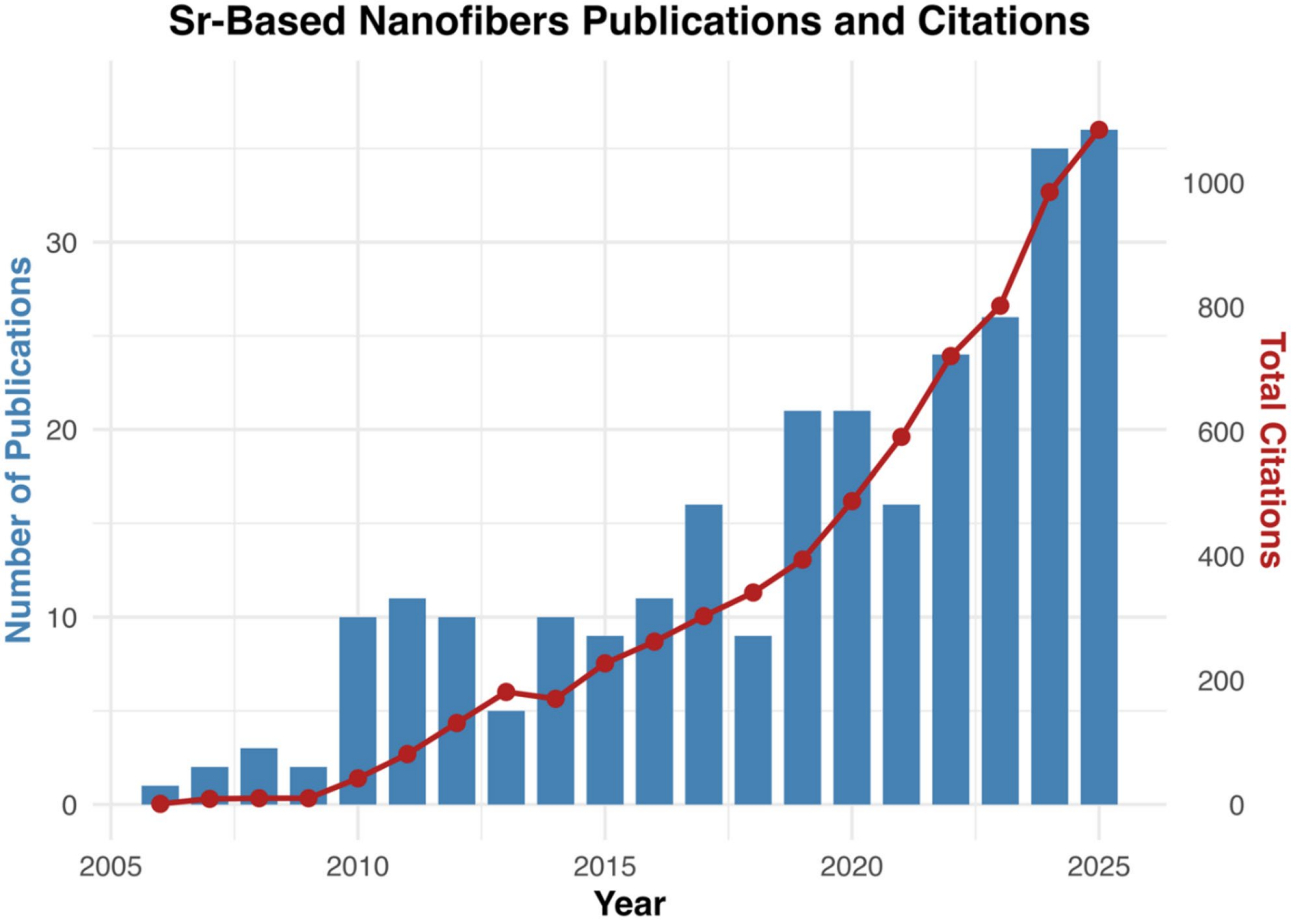
Publication trends of Sr-based nanofibers (2006–2025). Bar graph represents the annual number of publications, and the line graph represents citations over the same period. Data retrieved from Web of Science Core Collection using the keywords “Strontium Nanofibers” (search date: January 2026)

Bibliometric data on publications and citations were obtained from the Web of Science database on 06 January 2026. Data analysis and visualization were performed using R 4.4.1 (GUI 1.80 Big Sur ARM build 8416). This analysis demonstrates a clear upward trajectory in both research output and scientific impact over the past two decades. The field was first introduced in 2006, with only sporadic publications until 2009. A marked increase in output was observed in 2010, followed by steady annual contributions of approximately 8–10 studies through 2016. During this period, citations grew significantly, reflecting the recognition of Sr-based nanofibers as promising biomaterials, particularly for bone regeneration. From 2017 onwards, research activity accelerated, with annual publications rising to 20 by 2019–2020, paralleled by a sharp increase in citations exceeding 300. The most notable expansion occurred between 2021 and 2024, where publications nearly doubled from 16 to 34 per year, while citations surpassed 500. This rapid growth highlights the maturation of the field and the broadening of applications from bone repair to include skin, cartilage, tumor, and drug delivery. Collectively, these trends highlight the increasing global interest in Sr-based nanofibers and their transition from an emerging concept to a well-established research direction with considerable translational potential **(**Fig. 1**)**.

According to the best of our knowledge, there is no review that demonstrates the various techniques for the fabrication of Sr-based nanofibers and their potent effects in widespread biomedical applications. In this review, we will discuss the fabrication techniques of Sr-based nanofibers, their recent biomedical applications, challenges, and future perspectives.

## Fabrication techniques of Sr-based nanofibers

**Fig. 2:**
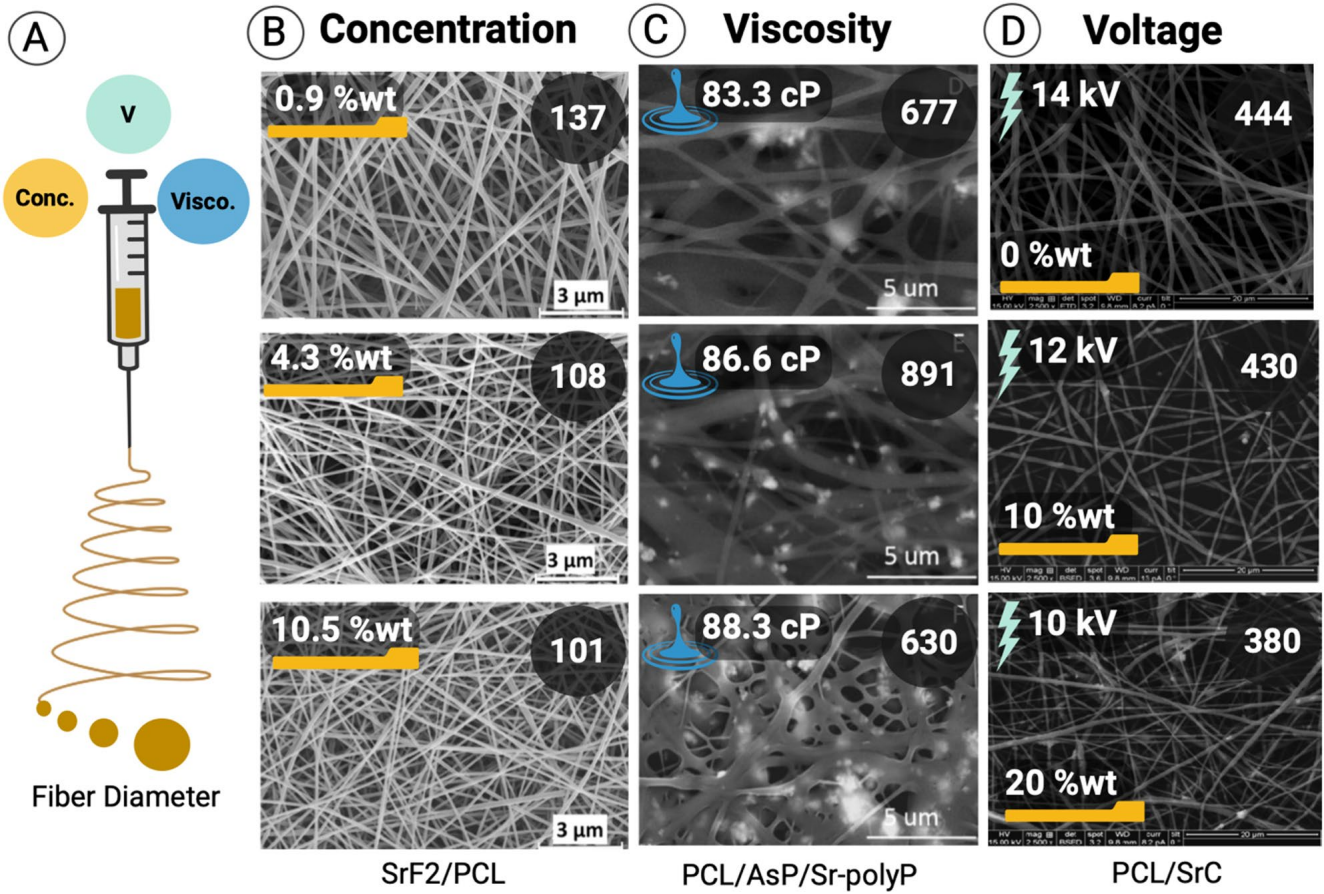
Effects of key electrospinning variables on fiber diameter in Sr-based nanofibers. (**A**) schematic diagram of variables influencing variations in fiber diameter. (**B**) Sr concentration: increasing Sr loading leads to larger fiber diameters (adapted with permission from Pishva et al., 2022 [48]; Copyright, Elsevier). (**C**) Viscosity: higher solution viscosity yields larger fiber diameters (reproduced from Abdel-Hady et al., 2024 [44]; under creative commons CC- by li-cense). (**D**) Applied voltage: decreasing the applied voltage reduces fiber diameter (adapted with permission from Meka et al., 2016 [53]; Copyright, Elsevier). Created in BioRender

**Fig. 3:**
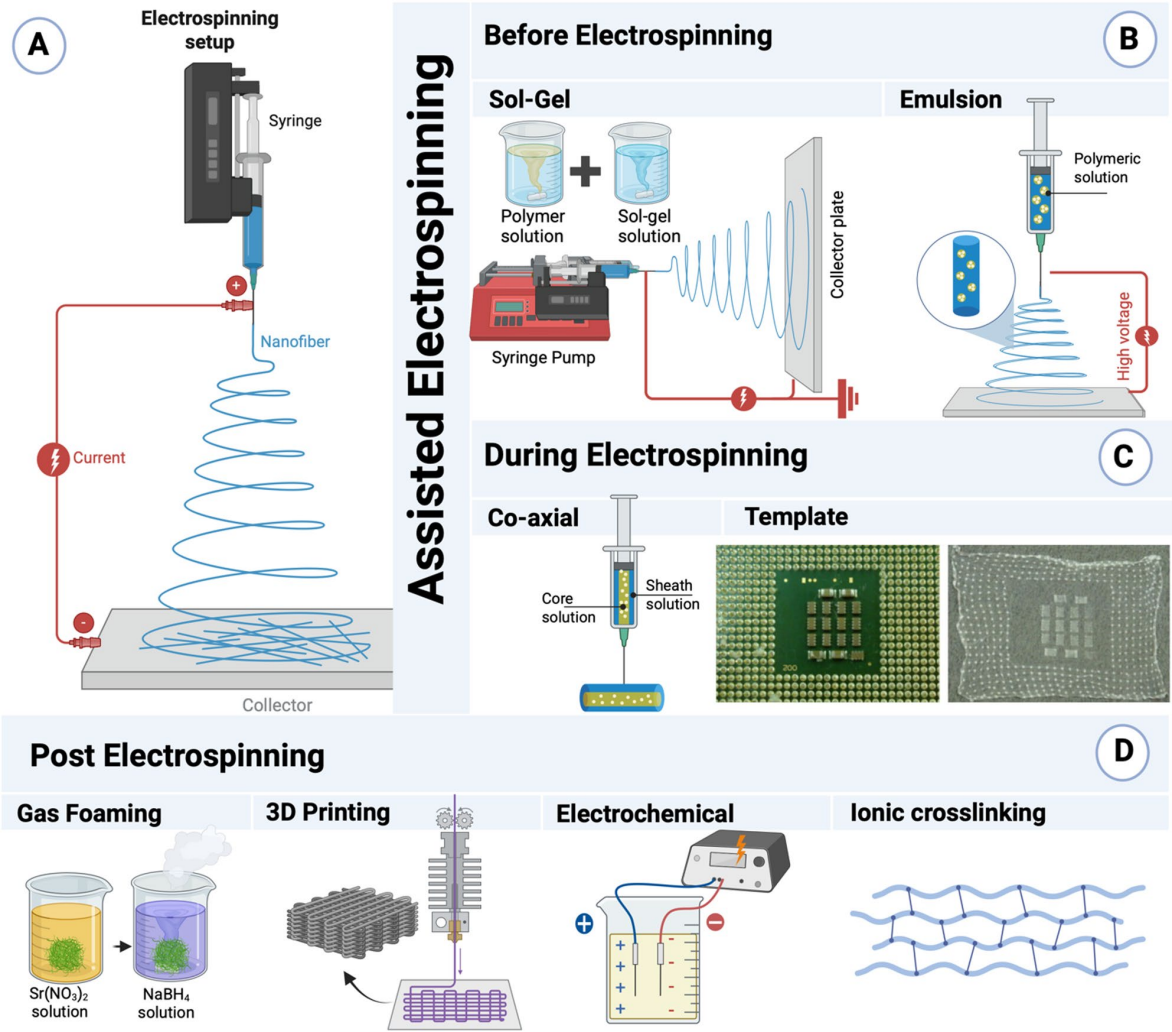
Schematic diagram of electrospinning and assisted electrospinning techniques. (**A**) Direct electrospinning setup. (**B**) Assisted before processing electrospinning modifications, such as sol–gel processing and emulsion electrospinning. (**C**) Assisted during processing electrospinning techniques, including co-axial and template electrospinning, that modify fiber morphology or enable core–shell structures. (adapted from Wu et al. [65]; copyright Elsevier). (**D**) Assisted Post processing electrospinning strategies to enhance structural or functional characteristics of the electrospun scaffolds. Diagrams were created with BioRender

**Fig. 4:**
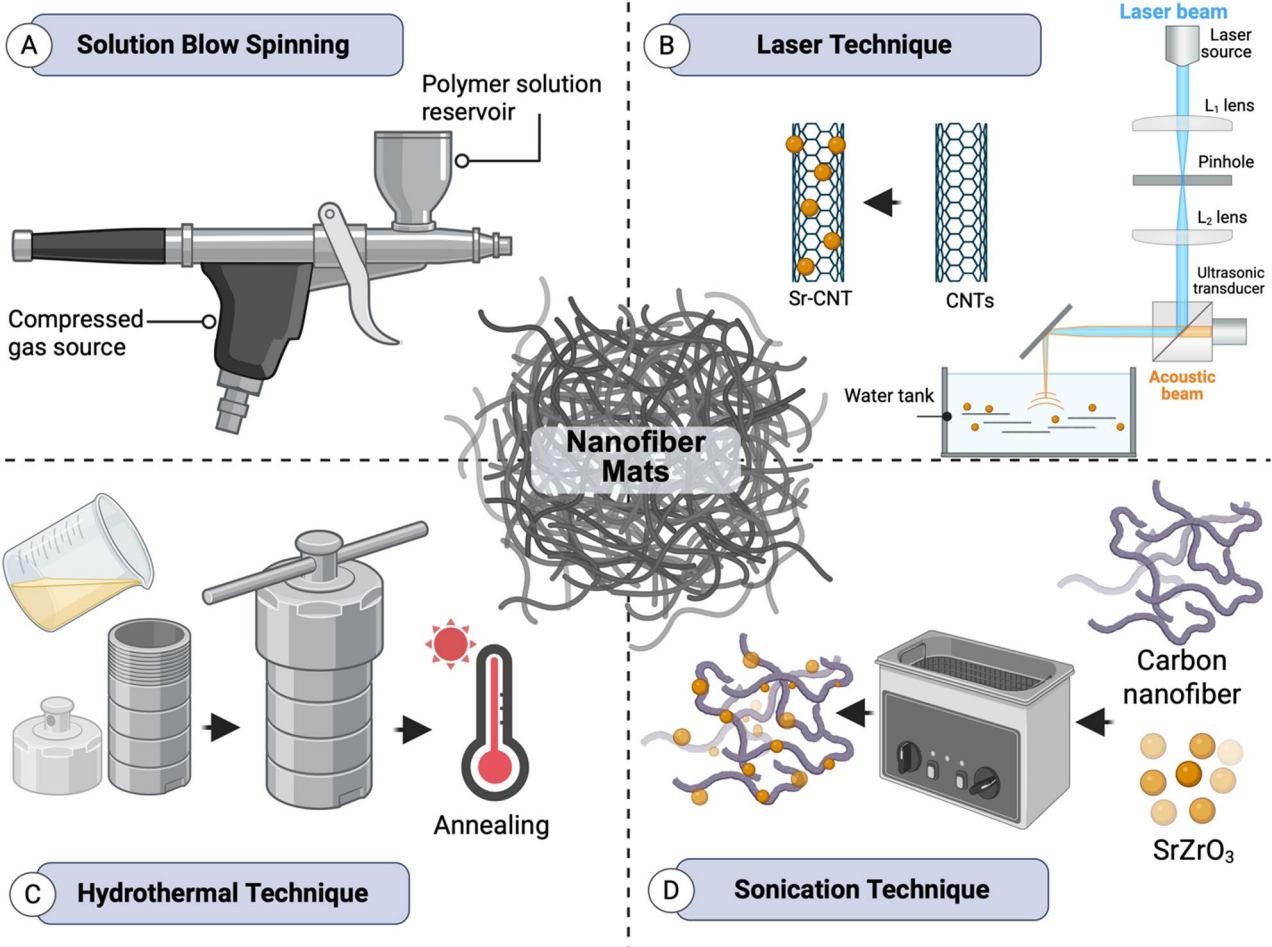
Schematic illustration of non-electrospinning techniques used for nanofiber and nanocomposite fabrication. (**A**) Solution blow spinning, in which a high-velocity gas stream stretches a polymer jet to form fine fibers. (**B**) Laser technique, where a focused laser beam ablates or melts the target material, generating nanoparticles or fibers that deposit onto a collector. (**C**) Hydrothermal technique, in which precursors react in a sealed autoclave under elevated temperature and pressure to produce crystalline or fibrous structures. (**D**) Sonication technique, where ultrasonic energy induces acoustic cavitation, promoting nanoparticle dispersion or assisting composite synthesis. All schematics were created using BioRender

Noteworthy, there are various techniques that have been used to fabricate Sr-based nanofibers according to the desired applications and features [37–40]. Each technique distinguishes with advantages, disadvantages and specific form of nanofibers. These techniques can be classified into three classes; (a) direct electrospinning techniques [41], where nanofibers are formed by both electrostatic and mechanical forces, (b) assisted-electrospinning techniques [42], where nanofibers are formed by two combined techniques, and (c) non-electrospinning techniques [43], where nanofibers are formed by mechanical forces only such as laser technique. The details fabrication techniques are summarized in Figs. 2, 3 and 4.

### Direct electrospinning technique 

Electrospinning is a widely used technique for fabricating ultrafine polymer fiber with tunable physiochemical properties [44]. It has emerged as a preferred method for producing Sr-based nanofibers due to its simplicity, cost-effectiveness, adaptability, and compatibility with a wide range of polymers (Figs. 2 and 3) [45–47]. The morphology and quality of electrospun Sr-based nanofibers are influenced by three main categories of parameters: polymer solution, processing, and environmental conditions (Fig. 2B–D) [25].

**Polymer solution parameters** (e.g., solvent dielectric properties, charge density, viscosity, surface tension, and conductivity) strongly affect fiber formation. For instance, Pishva et al. reported that incorporating strontium fluoride (SrF_2_) into polycaprolactone (PCL), reduced nanofiber diameter from 156 ± 61 nm (PCL alone) to 137 ± 35 nm, 108 ± 24 nm, and 101 ± 21 nm for 5, 10, and 15% SrF_2_, respectively, due to increased solution conductivity and charge density **(**Fig. 2B**)** [48, 49]. Similarly, Chen et al. showed that adding strontium oxide (SrO) to polylactic acid/gelatin (PLA/GEL), decreased the nanofiber diameter from 848 ± 180 nm to 614 ± 212 nm, 675 ± 245 nm, and 696 ± 290 nm at 0.25, 0.5, and 1% (w/v), respectively, whereas concentrations > 1% produced suspensions unsuitable for electrospinning [50]. Indong et al. further demonstrated that low Sr^2+^ concentrations (4–6%) supported continuous fiber formation, while higher levels (8–10%) increased surface tension, hindering nanofiber collection and leading to bead formation [51]. Interestingly, 4 and 6% of Sr-crosslinking also reduced the water uptake to 28.1% and 20.2%, respectively, consistent with previous findings [52]. Optimization of solution composition is therefore essential to minimize bead defects and obtain uniform fibers. For example, Abdel-Hady et al. successfully fabricated PCL/ascorbyl palmitate/Sr-polyphosphate nanofibers at 26 kV, showing that AsP or Sr-polyP increased fiber diameter by enhancing viscosity. The mechanical properties has improved with AsP up to 10%, but further additions of AsP or Sr-polyP reduced the mechanical strength **(**Fig. 2C**)** [44].

**Processing parameters**, including applied voltage, flow rate, tip-to-collector distance, and drum speed, also critically influence fiber morphology. Meka et al. fabricated electrospun Sr-carbonate-loaded PCL nanofibers (10 and 20% w/w) under various conditions (15 cm, 14 kV, 0.5 mL/h; 15 cm, 12 kV, 0.5 mL/h; and 12 cm, 10 kV, 0.5 mL/h), observing reductions in fiber diameter from 444 ± 171 nm (control PCL) to 428 ± 173 nm and 380 ± 200 nm for 10% and 20% Sr-carbonate, respectively **(**Fig. 2D**)** [53]. Higher particle loadings promoted aggregation and non-uniform stretching, further decreasing fiber diameter [54, 55]. One of the important parameters that affects the formation of nanofibers by electrospinning is the speed of the rotating drum [56]. The speed that matches the velocity of the rotating drum with evaporated jet depositions is called the alignment speed. When the rotating speed of the drum is higher than a specific limit, this will lead to the formation of discontinuous fiber due to the breakup of the fiber jet. On the other hand, when the rotating speed is lower than the alignment speed, fibers with fair alignment will be obtained [57]. To the best of our best knowledge, up to date there is no previous report that has demonstrated the effect of drum speed on the morphology of formed Sr-based nanofibers.

**Environmental parameters**, particularly humidity and temperature, can also affect electrospinning. Although no studies have specifically examined these effects in Sr-based nanofibers, related work on polyvinyl alcohol (PVA) fibers demonstrated significant changes in fiber diameter with humidity variations: 161 ± 42 nm at 60% relative humidity *versus* 667 ± 83 nm at 40% [58]. Increased humidity has generally been associated with smaller diameters and bead formation [59].

Despite its wide applicability, conventional electrospinning faces limitations, including poor cellular infiltration due to the dense fiber packing and small pore sizes, residual solvent toxicity, limited mechanical strength, low productivity, and high packing density [60–64]. Consequently, assisted or modified electrospinning techniques have been developed to overcome these challenges and enhance suitability for tissue engineering applications.

### Assisted electrospinning techniques

To overcome the limitations of conventional electrospinning, several assisted techniques have been developed. These can be applied either before processing electrospinning (such as sol–gel, and emulsion electrospinning), during processing electrospinning (such as coaxial, and template-assisted electrospinning) or post-processing electrospinning (such as gas foaming, 3D printing, electrochemical-assisted, ionic crosslinking, and calcination-assisted electrospinning) (Fig. 3).

#### Before processing electrospinning

These approaches introduce modifications during fiber formation to improve nanofiber morphology, composition, or functionality (Fig. 3B).

##### Sol-gel-assisted electrospinning

The sol–gel process is a bottom-up wet-chemical method used to fabricate nanoscale materials, especially metal oxides. Typically, precursor solutions undergo hydrolysis (water/alcohol) followed by condensation into a colloidal sol that evolves into a gel [66–70]. This sol–gel mixture is then blended with a polymer solution for electrospinning. Compared to conventional methods, sol–gel processing offers low cost, low-temperature processing, compositional control, high purity, and tunable porosity [66, 71–73].

Recent studies highlight the synergistic benefits of combining sol–gel and electrospinning. Zare et al. prepared Sr-containing bioactive glasses-PVA nanofibers, achieving coarse and uneven fibers with reduced diameters (275 ± 66 nm to 220 ± 36 nm) after calcination due to polymer removal indicating their conversion to Sr-containing bioactive glass nanofibers with the same composition of Sr^2+^ ions [38]. Similarly, Li et al. fabricated multiferroic polyvinyl pyrrolidone (PVP) nanofibers containing barium, strontium titanate (SrTiO_3_), and cobalt precursors. Heat treatment at 700 °C yielded rough, compact fibers with smaller diameters (100–400 nm) owing to PVP decomposition [74].

Despite its versatility, sol–gel electrospinning suffers from slow processing, low wear resistance, weak bonding, and sensitivity of precursors to moisture which limits large-scale applications [75].

##### Emulsion electrospinning

Emulsion electrospinning provides a simpler and more cost-effective route to core–shell fibers compared to coaxial methods. Herein, immiscible solutions (e.g., water and oil phases) are emulsified using surfactants before electrospinning. The distribution of phases determines the core–shell structure, and drug release can be tailored by adjusting viscosity and concentration [76–79]. Fiber morphology depends strongly on solution viscosity and composition. Abdollahi et al. fabricated strontium ranelate(Sr-Ran)-loaded fibers using PVA (core) and PCL (shell) [80]. Higher Sr-Ran concentrations increased fiber diameter due to higher viscosity, consistent with reports on inorganic-loaded fibers [80, 81]. Later, they demonstrated that higher Sr-Ran loading (20%) enhanced fiber degradation and water uptake due to reduced shell/core thickness ratios [80].

Unlike conventional electrospinning, both emulsion method preserve protein activity and 3D structure during encapsulation [82, 83]. Emulsion electrospinning is widely preferred due to mild solvent compatibility but requires careful emulsifier selection to maintain stability [83–85].

#### During processing electrospinning

In some cases, the fabrication of Sr-based nanofibers occur during electrospinning processes such as co-axial and template-assisted techniques (Fig. 3C).

##### Co-axial electrospinning

By contrast to emulsion electrospinning, coaxial electrospinning offers superior control over shell–core composition. The choice between them depends on the target application. Coaxial electrospinning enables the fabrication of core–shell nanofibers by feeding two solutions through concentric capillaries [82]. Typically, the shell consists of a polymer, while the core carries drugs or other active compounds. Electrostatic forces stretch the compound droplet into a coaxial jet, resulting in a core–shell nanofiber [86, 87]. This method allows precise control over drug release, reproducibility, and high-throughput fabrication. More complex multi-core structures can be produced by using additional syringes [69, 88].

Key parameters include flow rates, solution viscosity, and conductivity [82]. Wang et al. demonstrated the formation of hollow SrFe_12_O_19_ nanofibers by using PVP as a core and strontium nitrate (Sr(NO_3_)_2_)/Ferric nitrate (Fe(NO_3_)_3_)/PVP as a shell solution. After calcination, hollow fibers exhibited doubled surface area compared to conventional nanofibers, enhancing their potential for photocatalysis, sensing, and electromagnetic absorption [89–92]. Chen et al. reported PCL/PLGA shell fibers with PVA-Sr core, showing tunable degradation and sustained Sr release, with optimal core–shell formation at PCL: PLGA ratio of 1:1 [93]. The main limitation of coaxial electrospinning lies in its complex setup, requiring multiple pumps and coaxial needles.

##### Template-assisted electrospinning

In this method, patterned collectors or electrodes guide fiber alignment, producing organized meshes with defined pore structures [65, 94–96]. This enables fabrication of aligned or woven structures suited for micro-manufacturing. Xiao et al. combined template-assisted electrospinning with 3D printing to prepare Sr-containing scaffolds i.e., strontium hydroxyapatite (Sr-HAp). PCL/silk fibroin/Sr-HAp fibers were collected on a mesh template, layered with photo-cured hydrogel, and assembled into 3D constructs. The incorporation of Sr-HAp increased the fiber diameter from 735 nm to 823 nm, and enhanced cellular attachment and infiltration compared to random fibers [42, 97].

#### Post processing electrospinning

Post-treatment strategies further improve the architecture and biological performance of electrospun mats. Reported approaches for Sr-based nanofibers include gas foaming, 3D printing, electrochemical-assisted, ionic-crosslinking, and calcination-assisted techniques (Fig. 3D).

##### Gas-assisted technique

Gas foaming transforms 2D nanofibrous mats into 3D scaffolds with improved porosity and cell infiltration [98–101]. The process involves the immersion of electrospun mats in a foaming agent solution such as sodium borohydride (e.g., NaBH_4_), nucleation of hydrogen gas bubbles, and finally nanofiber expansion [31]. In addition, NaBH_4_ can reduce metal salts, simultaneously generating nanoparticles on fiber surfaces. For example, PCL mats immersed in Sr(NO_3_)_2_ followed by NaBH_4_ produced Sr-decorated scaffolds [31]. Recently, Chen et al. reported a significant increases in porosity and pore size, facility of cell infiltration and migration after gas foaming [102]. However, strong reducing agents may damage polymers or bioactive molecules, limiting applicability.

##### 3D printing electrospinning

3D printing provides customizable scaffold geometry, pore size, and mechanical properties at room temperature, allowing incorporation of heat-sensitive biomolecules [103]. Zhou et al. combined electrospinning with 3D printing to fabricate Sr-HAp@PCL integrated with PLGA/GEL nanofibers containing dimethyloxalylglycine-loaded mesoporous silica nanoparticles. Short nanofibers filled scaffold gaps, tuning porosity and mechanical properties [103]. Although effective for bone regeneration, challenges remain, including low mechanical strength and limited osteogenic performance of current 3D-printed composites.

##### Electrochemical-assisted technique

Pulse electrochemical technique is a surface modification method that induce redox reaction of electrolyte ions at cathode and anode poles, forming uniform distributed coating on the composite surface [104]. Direct methods such as electrospinning in which the metals were embedded inside the nanofibers could reduce their biological effects. The homogeneity and particles agglomeration of the metal coating could also be affected [105, 106]. By pulse electrochemical technique, metal ions can be coated on the surface with enhanced physiochemical and biological effects. Liu et al. [45] have prepared PLA/HAp composite nanofibers using electrospinning which furtherly coated with Sr-HAp/Cu/Polypyrrole (PPy) composite by pulse electrodeposition method. The main reason for using PPy is to regulate the deposition of Cu and Sr on the surface in a uniform way with a more significant amount of Sr-HAp. This strategy prevented any agglomeration on the surface and enhanced the osteogenic, angiogenic, and antibacterial effects of metal ions coated on the surface.

##### Ionic-crosslinking technique

Ionic crosslinking is a recent technique that enables bioactive materials to immobilize on the surface of electrospun membrane and exhibit their biological activity. In contrast, loading the bioactive materials within the membrane might reduce their effects. For example, Gönen et al. [107] incorporated both of Sr^2+^ and Cu^2+^ within the nanofiber where their osteogenic, angiogenic and antibacterial effects have weakened. On the other hand, Dodero et al. had fabricated alginate-based mats embedding zinc oxide nanoparticles (ZnO NPs) by electrospinning technique. The fabricated nanofibers were ionically crosslinked with bivalent ions (Ca^2+^, Sr^2+^ or Ba^2+^ ions) [108]. The nanofibers formed were smooth, homogenous, highly stable, high porosity, and well-defined after crosslinking with Sr^2+^ ions, with diameter 100 ± 30 nm. The mechanical properties of the Sr-crosslinked ZnO-embedded nanofiber were like that of human skin which could maintain the same time to absorb the exudate.

##### Calcination-assisted technique

This method is used to fabricate polymer free ceramic nanofibers through two consecutive steps. The first step includes the application of electrospinning was applied to fabricate continuous and solid nanofibers. The second step includes calcination of resulting nanofibers at elevated temperature to remove the polymer matrix, leaving behind ceramic nanofibers with well-defined structural properties [109]. Several recent studies have discussed this technique to form nanofibers with photocatalytic activity. For example, Barua et al. have fabricated SnO_2_ nanofibers with different concentrations (1%, 3%, and 5% of Sr ) by conventional electrospinning, after that they calcined the resulting nanofibers at 600 °C to obtain SnO_2_ nanofiber and removed the polymer and the solvents [109]. They noticed that diameter of undoped SnO_2_ nanofibers (259.04 ± 25.57 nm) had slightly increased with introducing Sr (1%) (288.69 ± 17.24 nm) with maintaining their continuous and smooth structure. By increasing the Sr concentration to 3% and 5%, the nanofiber diameters have reduced to 209.49 ± 15.29 and 148.72 ± 5.98 nm, respectively, which increased the surface area. They also observed that with increased Sr concentration, lattice strain and densification increased during calcination [110]. Also, Guo et al. had fabricated strontium doped-lanthanum cobaltite (LaCoO_3_) (La_1−x_Sr_x_CoO_3_ (x = 0, 0.1, 0.15, 0.2)) nanofibers *via* electrospinning to study the effect of doping Sr on the morphology and electrochemical properties [111]. They noticed that the nanofibers exhibited a robust hollow structure and reduced diameters with increased Sr content, resulting from lattice distortion [112]. La_0.85_Sr_0.15_CoO_3_ showed the smallest diameter, with an average size of 384 nm. Calcination increased the surface roughness of nanofibers, resulting in irregular fiber diameters with a nanotube-like shape due to decomposition of PVP. Similarly, a recent study demonstrated the fabrication of one-dimensional strontium rhodium oxide (Sr_6_Rh_5_O_15_) nanofibers by electrospinning followed by thermal annealing at 900 °C [113]. The fiber morphology exhibited notably rough surface morphology with high crystallinity even at high temperature which refers that this fiber has high durability even at harsh conditions.

### Non-electrospinning techniques

While electrospinning is the most common method to fabricate Sr-based nanofiber, it exhibits some drawbacks such as specialized equipment, high electrical potential, and electrically conductive collector [47]. Consequently, in recent years, novel strategies have been developed to fabricate Sr-based nanofibers with larger scales and high productivity such as solution blow spinning (SBS), laser-spinning, hydrothermal, and sonication techniques (Fig. 4).

#### Solution blow spinning technique

The solution blow spinning (SBS) technique is a novel technique used to fabricate fine fiber at high yield, with high surface area, allowing incorporation of therapeutic agents effectively and enhancing the bioactivity of biomaterials **(**Fig. 4A**)** [114]. Additionally, in contrast to electrospinning, SBS technique is a low-cost method which doesn’t require applied high voltage. Interestingly, air-heated SBS (A-HSBS) technique is the best choice to fabricate inorganic nanofiber from solutions with higher viscosity or ionic content [115]. Heating the air accelerates the solvent evaporation and promotes the formation of solid nanofiber without any defects, which are more common when evaporation is inadequate in conventional electrospinning [116, 117]. Ramalho et al. have demonstrated the effect of incorporation Sr^2+^ ions into ICIE16 bioactive glass on rheological properties and characteristics of formed nanofiber synthesized by A-HSBS technique [115]. The results demonstrated free beads nanofibers with small diameter. By increasing the doping concentration of Sr^2+^ ions to 3% in ICIE16 precursor solutions, the viscosity has increased to 1600 mPa whereas the shearing rate decreased. The divalent cation of Sr are capable of forming ionic bridges between polymer chains, consequently, the polymer matrix becomes more denser [118, 119]. Additionally, Sr-induced electrostatic potential which subsequently reduced repulsion forces between negatively charged chains of the polymer [120]. Due to the incorporation of 1, 2, and 3% of Sr^2+^ ions, the viscosity of the solution increased and consequently the diameter of undoped nanofiber increased from 410 ± 140 nm to a microfiber with sizes of 1340 ± 400 nm, 1030 ± 310 nm, and 1260 ± 310 nm, respectively. Mogharbel et al. have fabricated luminescent nanofibrous membranes with photochromic anticounterfeiting capability from strontium aluminate nanoparticles (Sr-Al NPs) with different conditions from recycled polycarbonate (PC) waste [121]. By increasing the concentration of Sr-Al NPs (0.01, 0.02, 0.05, 0.07, 1, 1.25, and 1.5%), the contact angle increased from 143.7 ^o^ to 155.7^o^, and the diameter increased from 280 nm to 420 nm. Similar study demonstrated improved hydrophobicity with change of water contact angle from 138.2^o^ to 144.5^o^ by increasing the concentration of Sr-Al NPs [122].

#### Laser-spinning technique

Laser-spinning technique is used to produce ultrafine materials containing Sr by high-power laser. In this method, a small volume of precursor material is quickly heated (in the range of 2000 K) and melted up by a high-power laser **(**Fig. 4B**)**. The precursor material, which is being irradiated, moves at a controlled speed related to laser beam allowing stable melting resulting in either complete or incomplete cut. Meanwhile, the gas jet applied direct molten material to the bottom of the cut, forming a viscous droplet. Furtherly, a gas jet causes cooling and propelling of viscous droplet resulting in its elongation and formation of glass nanofiber [123]. The nanofibers obtained by this method are amorphous, cylindrical, fully dense, solid, and completely separated forming easy-to-handle cotton-like mesh [123].

Echezarreta-López et al. have used this method to fabricate bioactive glass-based nanofibers doped with Zn and Sr as an essential metal ions for bacterial inhibition [123]. Laser-spinning process was applied by high power CO_2_ laser that emitted continuous laser radiation (2.5 KW) which directed precursor materials to fabricate nanofiber. The obtained nanofibers (with dimeters of 10 nm to 5 μm) were cotton-like mesh, uniformly fully dense cylindrical, solid, and completely separated. It was also observed the formation of spherical particles mixed with nanofibrous morphology because the surface tension causes break-up of viscous filament during elongation and cooling leading to spherical particles [124]. In addition, they noticed that the density of nanofiber had increased from 2 g.cm^− 3^ to 2.3 g.cm^− 3^ which is due to incorporation of Mg^2+^, Zn^2+^and Sr^2+^ ions in their composition.

#### Hydrothermal technique

Hydrothermal technique is one of the methods that had been used to fabricate Sr-based nanofiber without electrospinning **(**Fig. 4C**)** [125]. The principle of this method is based on using Teflon-lined closed stainless-steel autoclave, where hydrothermal reactions were carried out. The reactors were put into an oven with decided temperature and then taken out to cool naturally at room temperature after the reaction had occurred [126]. This method have some advantages such as low cost and simplicity [127]. Cao et al., had fabricated smooth with 6 μm in length SrTiO_3_ nanofibers *via* hydrothermal method through the reaction of titanium dioxide (TiO_2_) with 10 M of sodium hydroxide [125]. The surface area of the prepared sample was 14.38 m^2^/g, which indicate that SrTiO_3_ had mesoporous structure [128]. This high surface area promotes relatively-high distribution density of the active center, which enhance the catalytic activity for dye decomposition in water treatment [129]. Similarly, Yan et al. had fabricated SrTiO_3_ nanofibers through the reaction of TiO_2_·nH_2_O and strontium hydroxide (Sr(OH)_2_.nH_2_O) as a starting materials by hydrothermal method [126]. The formation of one-dimensional nanostructure of SrTiO_3_ nanofibers is formed through the strong alkaline treatment of TiO_2_·nH_2_O to form [Ti(OH)_6_]^2−^ which furtherly reacted with Sr(OH)_2_·nH_2_O at the hydrothermal conditions to form the nanofibers. In another study, heterostructures SrTiO_3_/TiO_2_ nanofibers was synthesized through the reaction of Sr(OH)_2_ with TiO_2_ nanofibers at hydrothermal autoclave conditions [130]. The nanocomposite is composed of SrTiO_3_ nanocubes (edge length: 120–180 nm), and TiO_2_ nanofibers (diameter: 200–300 nm), which enhanced the charge separation and photocatalytic activity in the decomposition of Rhodamine B under ultraviolet light. Increasing the concentration of precursor Sr(OH)_2_, increases the length and density of Sr(OH)_2_ nanocubes-based TiO_2_ nanofibers.

#### Sonication technique

Sonication techniques are used to uniformly distribute nanoparticles efficiently. In this technique, the prepared Sr-based nanoparticles are mixed with functionalized carbon nanofibers (F-CNF) under sonication which leads to efficiently fabricated Sr-based F-CNF composites **(**Fig. 4D**)** [131, 132]. Carbon nanofiber (CNF) is a novel approach defined as a standout from the graphitic framework, for enhanced electrical conductivity due to its hybridized sp^2^ carbon structure [133]. The CNF provided a large surface area with ample active site density for target species adsorption, which enhances the sensitivity of the sensor [134, 135]. Additionally, the flexible nature and high mechanical strength of CNF enable the proposed sensor with operational consistency and durability. For example, Sherlin et al. have demonstrated the fabrication of alkaline earth zirconates AZrO_3_ (A = Ca and Sr) incorporated into functionalized CNF and modified glassy carbon electrode (GCE) with them (SrZrO_3_/F-CNF/GCE) [131]. Compared to CaZrO_3_/F-CNF nanocomposite, SrZrO_3_/F-CNF nanocomposite has reduced the average crystallite size of the nanocomposite, indicating an increase in the surface area, which was essential for improving the performance of the SrZrO_3_/F-CNF in electrochemical sensing applications. Also, Bharathi et al. have exhibited the fabrication of Sr_2_P_2_O_7_@F-CNF to induce their effect and conductivity [132]. The formed nanofiber was interconnected with one another and formed a network-like structure that is favorable for better conductive and sensing performance (Table 1).

**Table 1:** Factors affecting the properties of Sr-based nanofibers fabricated by direct electrospinning, assisted before electrospinning, assisted during electrospinning, assisted post-electrospinning, and non-electrospinning techniques

Item	Composite	Change	Effects	Ref
***Direct Electrospinning***				
**Electrospinning**	PS	Incorporation of SrF_2_ into PCL	• The charge density has increased. • The conductivity has increased. • The nanofiber diameter has reduced. • Crystallinity has increased.	[48]
	PLA/GEL/SrO	Incorporation of SrO into PLA/ GEL	• The solution viscosity has reduced. • The nanofiber diameter has reduced. • Any addition of SrO more than 1% (wt/v) can’t form a nanofiber.	[50]
	PCL/AsP/Sr-polyP	Incorporation of ascorbyl palmitate and strontium polyphosphate	• The polymer solution viscosity has increased from 40.6 ± 2.5 to 66.6 ± 2.0 cP. • Conductivity decreased from 7.3 ± 0.4 to 6.38 µs and then increased with high Sr-polyP contents. • The nanofiber diameter has increased. • The mechanical properties have increased till 10% of Sr-polyP, then mechanical properties decreased. • The water contact angles have decreased.	[44]
	TPU/Sr-HAp	Incorporation of Sr-HAp nanorod (1, 3, 5, 7 wt%)	• The solution charge density has increased by adding Sr-HAp of 1, 3, and 5 wt%. • The nanofiber diameter has reduced by increasing the contents of Sr-HAp from 1–5 wt%. • The nanofiber diameter has increased when Sr-HAp was 7 wt%. • The water contact angle, porosity, roughness and mechanical properties have increased.	[136]
	PVA/SG/Sr-Ber-CQD	Incorporation of Sr.Ber-CQD with 1, 3, 5, and 10%	• The diameter of the nanofibers has increased as the percentage of Sr-Ber-CQD increased. • Nanofibers with 3 and 5% Sr-Ber-CQD had the greatest swelling capability. • The water vapor permeability rate has increased when the percentage of Sr-Ber-CQD was increased.	[137]
	PCL/SrCO_3_	Strontium carbonate incorporated into PCL with changed processing conditions (distance and voltage)	• Change processing conditions (15 cm, 14 kV, 0.5 ml/h) for PCL, (15 cm, 12 kV, 0.5 ml/h) for PCL/SrC10, and (12 cm, 10 kV, 0.5 ml/h) for PCL/SrC20. • The formed nanofibers have a similar range of diameters. • High content of loading particles of Sr led to non-uniform nanofiber with reduced diameter.	[53]
***Assisted before-electrospinning***				
**Sol-gel**	BST/PVP	Calcination temperature	• Significant weight loss was observed below 350 °C, indicating removal of solvents. • Calcination between ∼330 and 380 °C indicated decomposition of TIAA along with the degradation of PVP. • Calcination at 580 °C indicated decomposition of main chain of PVP and the formation of metal oxide phase of perovskite BST.	[138]
	BSTCO/PVP	Calcination temperature	• Calcination at 388 °C and 490 °C indicated the decomposition of PVP and burning of decomposed carbon. • BSTCO/PVP composite nanofibers without heat treatment exhibited smooth surface, and a uniform diameter of 200–500 nm over their length. • Unlike the BSTCO, nanofibers annealed at 700 °C showed rough surface morphology and relatively tight structure.	[74]
**Emulsion**	PVA-Sr-Ran/PCL	Incorporation of Sr-Ran	• The viscosity of the solution has increased. • The nanofiber diameter has increased. • The water contact angle has reduced. • The mechanical strength has enhanced.	[39]
	PCL-PVA/Sr-Ran	Incorporation of Sr-Ran into PVA core (0.05 and 0.2%), and PCL as shell	• The viscosity of core solutions has increased with increasing the amount of Sr-Ran. • The hydrophilicity of nanofiber has increased, with increasing the amount of Sr-Ran. • The nanofiber diameter has increased with increasing the amount of Sr-Ran. • The water uptake and degradation have increased with increasing the amount of Sr-Ran.	[80]
***Assisted during-electrospinning***				
**Coaxial**	PVP@PVP/[Sr (NO_3_)_2_+Fe(NO_3_)3.9H_2_O]	Calcination temperature	• Minor weight loss (~ 5.78%) was below 230 °C due to removal of DMF from the system. • Steep weight loss (~ 69.70%) between 230 °C and 380 °C indicated decomposition of PVP. • Slow weight loss (~ 5.94%) in the range of 380 °C to 450 °C indicated decomposition of PVP, Sr and Fe nitrates. • No weight loss above 450 °C demonstrated the formation of decomposition products of the SrM.	[89]
	Sr^2+^-doped PCL/PLGA -PVA NFs.	Loading SrCl_2_ in both core and shell .	• The viscosity of core solutions has increased. • Formation of clear core-sheath structure nanofiber with diameter ratio (4:1) of core to sheath. • The porosity of Sr^2+^-doped (64.34%) was obviously higher than those of NFs without Sr^2+^-doping (52.87%).	[93]
	PCL/Sr-HAp/DFO	Loading DFO in the core and Sr-HAp in the shell	• The fiber showed smooth and uniform distribution. • The diameter of the fiber increased by adding DFO and/or Sr-HAp. • No effect for Sr-loading on the water contact angle or stiffness of the nanofibers.	[139]
**Template-assisted method**	Mesh@Sr-HAp	Incorporation of Sr-HAp into	• The viscosity has increased, which affects stretchability. • The nanofiber diameter has increased from 735.3 ± 55.1 nm to 823.2 ± 91.8 nm.	[42]
***Assisted post-electrospinning***				
**Gas foaming**	3DS-Sr	Incorporation of Sr(NO_3_)_2_	• High concentrations (20, 200 mM) are preferable. • The viscosity has increased • The nanofibers were formed with less change in thickness, volume, density, and porosity. • The nanofiber diameter has increased. • The mechanical properties were slightly enhanced.	[31]
	3DS-E	Modification of metal phenolic networks	• The presence of clustered particles on both the surface and cross-section. • The nanofiber diameters slightly increased from 461 ± 138 nm to 497 ± 100 nm • The inter-fiber gaps, pore size, and porosity have slightly reduced.	[102]
**3D printing**	DMSNs/Sr-HAp@PGP	Incorporation of Sr-HAp	• The morphology has changed. • The nanofiber diameter has decreased. • The nanofiber roughness has increased. • The nanofiber became denser, and pore size has obviously decreased.	[103]
**Electrochemical method**	PLA/HA@Sr-HAp/Cu/PPy	strontium-doped HAp coating in the PPy on the nanofiber surface	• The surface energy has increased • The diameter of the nanofiber has increased. • The water contact angle has reduced.	[45]
**Ionic crosslinking**	Alg-ZnO/Sr^2+^	Alginate mats crosslinked with Sr^2+^	• The resultant nanofibers were homogeneous and smooth with 100 ± 30 nm dimeter. • The mechanical properties of the nanofibers had enhanced. • The water vapor permeability of Sr-crosslinked alginate nanofiber 3.8 × 10^− 12^ g/mPa s.	[108]
**Calcination-assisted**	SnO_2_/Sr nanofibers	Doping SnO_2_ with 1, 3, and 5% of Sr followed by calcination at 600 °C	• The SnO_2_ nanofiber diameter increased by doping with 1% of Sr content, whereas it reduced when 3, and 5% of Sr was used. • The fiber uniformity was improved with increasing the doping concentration of Sr. • 1% Sr-doped SnO_2_ nanofibers exhibited the highest photocatalytic activity for methylene blue under both UV and visible light with 89% and 84.74%, respectively.	[109]
	La_1−x_Sr_x_CoO_3_	Calcination temperature at 700 °C	• Calcination caused irregular fiber diameters, increased the roughness of the surface, and reduced the fiber diameter. • The formed nanofibers showed uniform filamentous morphology with hollow nanotube structure after calcination. • La_0.85_Sr_0.15_CoO_3_ nanofiber has the lowest nanofiber diameter (75 nm). • La_0.85_Sr_0.15_CoO_3_ demonstrated specific capacitance of 265.5 F/g, with the lowest charge transfer resistance.	[111]
***None-electrospinning Techniques***				
**Solution blow spinning**	ICIE16-BG/Sr	incorporation Sr^2+^ ions into ICIE16 bioactive glass	• The viscosity has increased to 1600 mPa by increasing Sr^2+^ ions to 3% whereas the shearing rate decreased. • Free beads nanofibers. • The fiber diameter has increased from undoped nanofiber 410 ± 140 nm to microfibers with sizes of 1340 ± 400 nm, 1030 ± 310 nm, and 1260 ± 310 nm for of 1, 2, and 3% of Sr^2+^ ions.	[115]
	Sr-Al@PC	Incorporation of SrAl_2_O_4_:Eu^+ 2^, Dy^3+^ into PC waste	• The water contact angles of the Sr-Al@PC nanofibrous membranes have increased from 143.7^o^ to 155.7^o^ with the increased Sr-Al NPs content indicating better superhydrophobicity. • The Nanofiber diameter ranges between 280 and 420 nm. • Increasing the Sr-Al NPs led to an increase in Young’s modulus and tensile strength of the composite nanofibers.	[121]
	Sr-Al@PLA	Incorporation of SrAl_2_O_4_:Eu^+ 2^, Dy^3+^ into PLA nanofiber	• The viscosity of polymer solution has increased with increased Sr-concentration. • The water contact angle has increased from 138.2^o^ to 144.5^o^, indicating better superhydrophobicity. • The morphology of the composite nanofibers was like Sr-Al free samples which means that Sr-Al NPs are completely embedded inside the polylactic acid nanofiber. • The nanofiber diameter ranged in size from 100 to 220 nm.	[122]
**Laser-spinning**	Sr^2+^ and Zn^2+^ doped BG- nanofiber	-----	• The surface tension caused the break-up of the viscous filaments. • Cotton-like mesh, uniformly fully dense cylindrical, solid, and completely separated nanofiber with a diameter of 10 nm to 5 μm. • Spherical particles formed with nanofiber. • The density of the nanofiber had increased from 2 g/cm³ to 2.3 g.cm^-3^.	[123]
**Hydrothermal**	SrTiO_3_ nanofibers	Synthesis of SrTiO_3_ nanofibers using hydrothermal method at 210 °C	• The resulting nanofibers showed smooth and fibrous structure, with about ∼6 μm in length. • SrTiO_3_ nanofibers showed high crystallinity with mesoporous structure. • Surface area of sample is 14.38 m^2^/g, a high specific area was related to high distribution density of active center, which enhanced the catalytic dye decomposition.	[125]
	SrTiO_3_/TiO_2_ nanofibers	Synthesis of nanocomposite nanofibers at Hydrothermal temperature of 150 °C with two different Sr(OH)_2_ concentrations	• High uniform and regular shape of SrTiO_3_ nanocubes were implanted on the primary TiO_2_ nanofibers. • The density of SrTiO_3_ nanocubes was dramatically increased when the Sr(OH)_2_ concentration increased by 10 times. • The length of SrTiO_3_ nanocubes changed from 150 to 250 nm with increased the concentration of Sr(OH)_2_. • Decreasing the autoclave temperature from 150 to 120 °C changes the SrTiO_3_ morphology from nanocubes to nanoparticles (crystal size: 40 × 60 nm).	[130]
**Sonication**	SrZrO_3_/F-CNF	Incorporation of SrZrO_3_ into functionalized caron nanofibers (F-CNF)	• The TEM analysis demonstrated combined structure of carbon nanofiber and the irregular shape of SrZrO_3_ without aggregation. • The resulting nanofibers increases the unmasked redox sites and assures rapid penetration of electrolytes which improves the electrochemical performance of the nanocomposite. • Modifying the GCE electrode using SrZrO_3_/F-CNF reduced the charge transfer resistance (R_ct_) value to the lowest of 306.48 Ω·cm^2^ which increased electrical conductivity.	[131]
	Sr_2_P_2_O_7_@F-CNF	Incorporation of Sr_2_P_2_O_7_ into functionalized caron nanofibers (F-CNF)	• The SEM analysis showed an interconnected fibers to form a network-like structure with size lower than 1 μm. • Several F-CNFs are aggregates on Sr_2_P_2_O_7_ surface. • Sr_2_P_2_O_7_@F-CNF showed a huge active surface area (A = 0.197 cm^2^). • Modifying the GCE electrode using Sr_2_P_2_O_7_/F-CNF reduced the R_ct_ value of 33.30 Ω·cm^2^. • The Sr_2_P_2_O_7_@F-CNF /GCE has the highest conductivity of 12.60 µA toward flufenamic acid with high selectivity and stability.	[132]

PS-G, polycaprolactone-based nanofiber incorporated with strontium fluoride and immobilized with gallic acid; PLA/GEL/SrO, strontium oxide particles into poly(L-lactic acid) and gelatin nanofibers; PCL/AsP/Sr-polyP, ascorbyl palmitate–polycaprolactone fiber mats loaded with strontium polyphosphate nanoparticles; TPU/Sr-HAp, thermoplastic polyurethane elastomer dispersed with Sr-HAp nanorods.; PVA/SG/Sr-Ber-CQD, strontium-doped berberine carbon quantum dots incorporated into polyvinyl alcohol and scleroglucan-based nanofiber; PCL/SrCO_3,_ strontium carbonate incorporated into polycaprolactone; BST/PVP, barium strontium titanate/polyvinyl poly(vinylpyrrolidone)-based nanofibers; BSTCO/PVP, CO-doped barium strontium titanate cobalt/ poly(vinylpyrrolidone)-based nanofibers; PVA-Sr-Ran/PCL, strontium ranelate/polyvinyl alcohol and polycaprolactone-based nanofibers; PVP@PVP/[Sr(NO_3_)_2_+Fe(NO_3_)_3_.9H_2_O], strontium nitrate and Ferric nitrate/polyvinyl poly(vinylpyrrolidone)@ polyvinyl poly(vinylpyrrolidone) core-shell composite nanofibers; Sr^2+^-doped PCL/PLGA-PVA NFs, strontium-doped polycaprolactone/poly (D, L-lactide-co-glycolide)-polyvinyl alcohol; PCL/Sr-HAp/DFO, polycabrolactone loaded with strontium-doped hydroxyapatite and desferrioxamine; Mesh@Sr-HAp, strontium-hydroxyapatite-enriched polycaprolactone/silk fibroin nanofibers; 3DS-Sr, 3D nanofiber scaffolds decorated with strontium nanoparticles; 3DS-E, 3D scaffold modified with metal phenolic networks composed of epigallocatechin gallate and Sr^2+^ ions; DMSNs/Sr-HAp@PGP, short nanofibers containing dimethyloxalylglycine-loaded mesoporous silica nanoparticles with a 3D printed strontium-contained hydroxyapatite/polycaprolactone scaffold; PLA/HA@Sr-HAp/Cu/PPy, polylactic acid/hydroxyapatite nanofiber coated with strontium-doped hydroxyapatite/copper/polypyrrole composite.; Alg-ZnO/Sr^2+^, Pure alginate and pure alginate with zinc oxide nanocomposite crosslinked with strontium; SnO_2_/Sr nanofibers, strontium-doped tin oxide-based nanofibers; La_1−x_Sr_x_CoO_3_, strontium doped-lanthanum cobaltite; ICIE16-BG/Sr, Strontium doped-ICIE16 bioactive glass; Sr-Al@PC, strontium aluminate nanoparticles/recycled polycarbonate; Sr-Al@PLA, Strontium aluminate nanoparticles/recycled polylactic acid; Sr and Zn-doped BG nanofibers, strontium and zinc-doped bioactive glass; SrTiO_3_, strontium titanate; SrTiO_3_/TiO_2_, strontium titanate/titanium dioxide; SrZrO_3_/F-CNF, strontium zirconate AZrO_3_/functionalized carbon nanofiber; Sr_2_P_2_O_7_@F-CNF, strontium phosphate/functionalized carbon nanofiber composite

## How strontium works on the biological side

Before discussing specific biomedical applications and their effectiveness in regenerating tissues and repairing defects, it is important to first understand the underlying mechanisms by which Sr-based nanofibers exert their biological effects.

### Anti-inflammatory and immunomodulatory effects

Inflammation is a critical factor influencing bone healing, implant integration, and tissue regeneration. Persistent inflammatory responses can impair angiogenesis, delay osteogenesis, and compromise biomaterial performance. Sr-enhanced nanofiber constructs represent a compelling avenue for immune regulation in tissue engineering. Though direct studies remain limited, parallels with Sr-integrated coatings and hydrogels illuminate a framework for design and function.

For example, Sr-Ran-infused lactoferrin-loaded titanium coatings effectively shifted macrophage polarization to M2, reducing inflammation while promoting angiogenesis and osteogenesis [140]. Similarly, Sr-Ran-laden keratin–hyaluronic acid (K/HA/Sr-Ran) hydrogels markedly reduced inflammatory mediators (interleukin-6 (IL-6), tumor necrosis factor-α (TNF-α), ROS, favoring wound regeneration [141]. In addition, green-synthesized SrO NPs exhibited in vitro anti-inflammatory activity by inhibiting protein denaturation [142]. Clinically, Sr-Ran use in osteoarthritis patients attenuated cartilage loss and bone marrow lesion progression, underscoring its systemic disease-modifying and inflammation-controlling potential [143].

Furthermore, Sr has been shown to exert potent anti-inflammatory effects by directly modulating canonical signaling cascades across skeletal and non-skeletal systems. In wear-particle–induced aseptic loosening, Sr^2+^ ions suppressed titanium particle-driven osteoclast activation and chronic inflammation through inhibition of nuclear factor kappa-light-chain-enhancer of activated B cells (NF-*κB)* pathway, resulting in reduced osteoclastogenesis, inflammatory cell infiltration, and bone loss, along with downregulation of receptor activated of nuclear factor *κB* ligand (RANKL), TNF-α, interleukin-1β (IL-1β), and IL-6 in a dose-dependent manner [144]. Similarly, Tan et al. [145] demonstrated that Sr attenuated liposaccharide-induced inflammation in bovine ruminal epithelial cells by suppressing the TLR4/MyD88/NF-κB pathway, decreasing pro-inflammatory cytokine production and phosphorylation of p65 and inhibitor of κB.

Meanwhile, polymeric nanofiber dressings combining β-glucan and chitosan demonstrated robust, concentration-dependent suppression of macrophage-derived nitric oxide, far exceeding the effect of either component alone, suggesting that bioactive fiber formulations can synergies to attenuate inflammatory responses [146]. By extension, embedding Sr (e.g., SrO, Sr-Ran, or ionic Sr) into electrospun nanofibers, possibly in conjunction with biopolymers like chitosan or β-glucan, offers a multifaceted strategy delivering immunomodulatory cues, dampening oxidative stress, and supporting tissue regeneration. Future investigations should focus on such composite nanofibers’ fabrication, release kinetics, macrophage modulation, ROS neutralization, and in vivo efficacy in inflammatory, osteogenic, or wound healing models.

In addition to preclinical and clinical evidence, the anti-inflammatory potential of Sr compounds has been recognized through several patented formulations. The patent EP1605955A2 [147] describes a therapeutic method for treating inflammation in human and non-human subjects by administering physiologically tolerable Sr compounds, targeting both pain-associated and non-pain-associated conditions. Complementing this, the patent ES2553107T3 [148] introduces a topical formulation with Sr, a biocompatible vehicle, and a penetration enhancer to manage subdermal and joint-related inflammation. More recently, the patent US20170049807A1 [149] discloses Sr–β-hydroxybutyrate–based compositions for wound management, pain, pruritus, and tissue irritation, underscoring the broad therapeutic versatility of Sr.

### Antioxidant effect

Oxidative stress is a major contributor to delayed healing and tissue degeneration, and materials with intrinsic or synergistic antioxidant activity are of great value in biomedical applications. Sr-based nanofibers have been explored as multifunctional systems that not only support regeneration but also provide antioxidant protection. For instance, PCL/AsP/Sr-polyP were shown to combine the osteogenic and regenerative potential of Sr with the free radical scavenging properties of AsP, resulting in an antioxidant platform suitable for guided bone regeneration [44]. Similarly, the development of PVA/SG/Sr-Ber-CQD nanofibrous dressing demonstrated enhanced antioxidant activity, which contributed to accelerate wound healing in animal models by mitigating oxidative stress–induced tissue damage [137]. Beyond biomedical regeneration, Sr-based nanofiber composites have also been engineered for sensitive detection of antioxidant molecules. Anchoring nanoflakes morphology of Sr-Al NPs on functionalized carbon nanofibers enabled efficient amperometric detection of the food additive propyl gallate, reflecting the strong electron-transfer and radical-interaction capacity of Sr-containing nanostructures [150].

### Angiogenesis and vascularization support

**Fig. 5:**
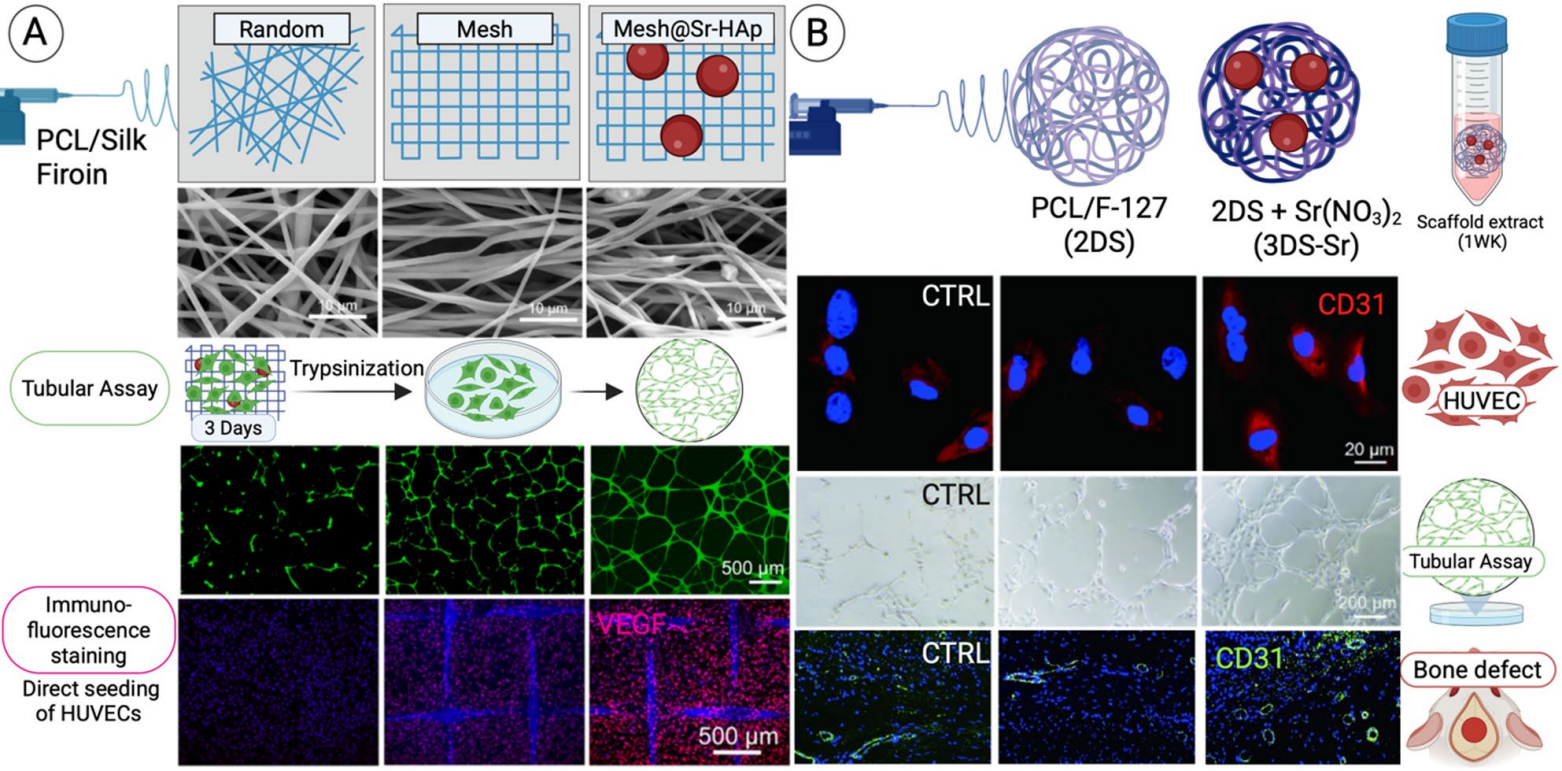
Angiogenesis activity of Sr-based nanofibers. (**A**) SEM images of electrospun PCL, silk fibroin, and Sr-HAp nanofibers. Tubular assay performed using HUVECs cultured for 3 days on materials, then trypsinized and assessed. Direct HUVEC seeding and VEGF staining after 3 days. Adapted with permission from Xiao et al. [42]. Copyright Springer Nature. (**B**) PCL/F127–Sr(NO₃)₂ scaffold extracts obtained by 1-week culture in medium, tested in vitro with HUVECs for 5 days (CD31 staining and tubular assay). In vivo angiogenesis assessed in a calvarial bone defect model with histological CD31 staining. Adapted with permission from Chen et al. [31]. Copyright ACS Applied Materials & Interfaces. Created in BioRender

Angiogenesis is essential for proper wound healing, as it ensures adequate nutrient and oxygen delivery to regenerating tissues [151]. Recent studies have demonstrated that Sr²⁺ enhances the secretion of pro-angiogenic factors and supports angiogenesis [152, 153]. In addition, Sr²⁺ ions improves the viability of fibroblasts, endothelial cells (HUVEC), and smooth muscle cells key players in blood vessel formation [154]. For instance, Zhang et al. reported that Sr-functionalized gelatin hydrogels promoted endothelial progenitor cell-driven angiogenesis, thereby enhancing wound tissue regeneration [155]. Similarly, Xiao et al. showed that Sr-HAp nanofibers with a mesh-like morphology exhibited a strong angiogenic effect **(**Fig. 5A**)** [42]. In their study, HUVEC migration was significantly enhanced in the Mesh@Sr-HAp nanofiber group compared to other scaffolds, leading to accelerated wound closure. Tube formation assays further confirmed that Mesh@Sr-HAp nanofibers most effectively supported endothelial function, enabling the development of continuous tubular networks. Immunofluorescence staining revealed elevated expression of HIF-1α and VEGF in HUVECs cultured on Mesh@Sr-HAp scaffolds, indicating increased microvascular permeability and endothelial proliferation, both of which contribute to angiogenesis [155].

In another study, Chen et al. demonstrated that Sr NPs-decorated 3D nanofiber scaffolds (3DS-Sr) markedly upregulated angiogenesis-related genes, including CD31, VEGF, and HIF-1α, compared to controls **(**Fig. 5B**)** [31]. Tube formation assays revealed that the 3DS-Sr group generated the most extensive tubular networks, characterized by increased numbers of nodes, meshes, and elongated branches. Protein analyses further confirmed higher levels of VEGF, CD31, HIF-1α, and phosphorylated ERK in the 3DS-Sr group. This effect was attributed to Sr-mediated activation of the ERK pathway, which in turn promoted the expression of angiogenesis-related markers and enhanced endothelial tube formation.

### Antimicrobial properties

Microbial infection represents a critical barrier to the success of many biomedical implants and scaffolds, as it can compromise healing, delay tissue integration, and increase healthcare burden [156]. Sr^2+^ ions has recently attracted attention for its intrinsic antibacterial and antimicrobial effects, making Sr-containing nanofibers promising candidates for a wide range of biomedical applications.

Several studies have demonstrated that Sr incorporation into nanostructured biomaterials can suppress bacterial adhesion and proliferation, thereby limiting biofilm formation. The release of Sr²⁺ ions have been shown to interfere with key bacterial processes such as cell wall integrity, DNA replication, and metabolic activity [157]. Importantly, this antibacterial potential has been reported across different material systems, including polymers, ceramics, and composites [158–160].

For example, Pishva et al. observed that SrF_2_–containing scaffolds significantly reduced bacterial optical density, preventing *P. aeruginosa* biofilm formation [48]. Similarly, Diputra et al. reported that while HAp alone showed no inhibition against bacterial growth, substitution with Sr conferred antibacterial properties against *E. coli* and *S. aureus*, with the effect increasing in a concentration-dependent manner [158]. Other studies support this trend, highlighting that Sr-substituted materials consistently displayed enhanced antibacterial performance compared to their non-substituted counterparts [159, 160].

Nevertheless, the antimicrobial efficacy of Sr is not universal. For instance, Sr released from resin-modified glass ionomer cements did not demonstrate significant antibacterial activity against cariogenic oral bacteria [160]. This suggests that the antimicrobial outcome depends strongly on factors such as Sr concentration, chemical form (e.g., SrF₂, Sr-HAp), and the targeted bacterial species.

Overall, Sr and Sr-containing nanofibers show considerable promise as dual-functional agents that can reduce the risk of infection while simultaneously supporting tissue integration.

### Osteo-immunomodulatory and bone-related cellular mechanisms

#### Stem cell proliferation and osteogenesis

**Fig. 6:**
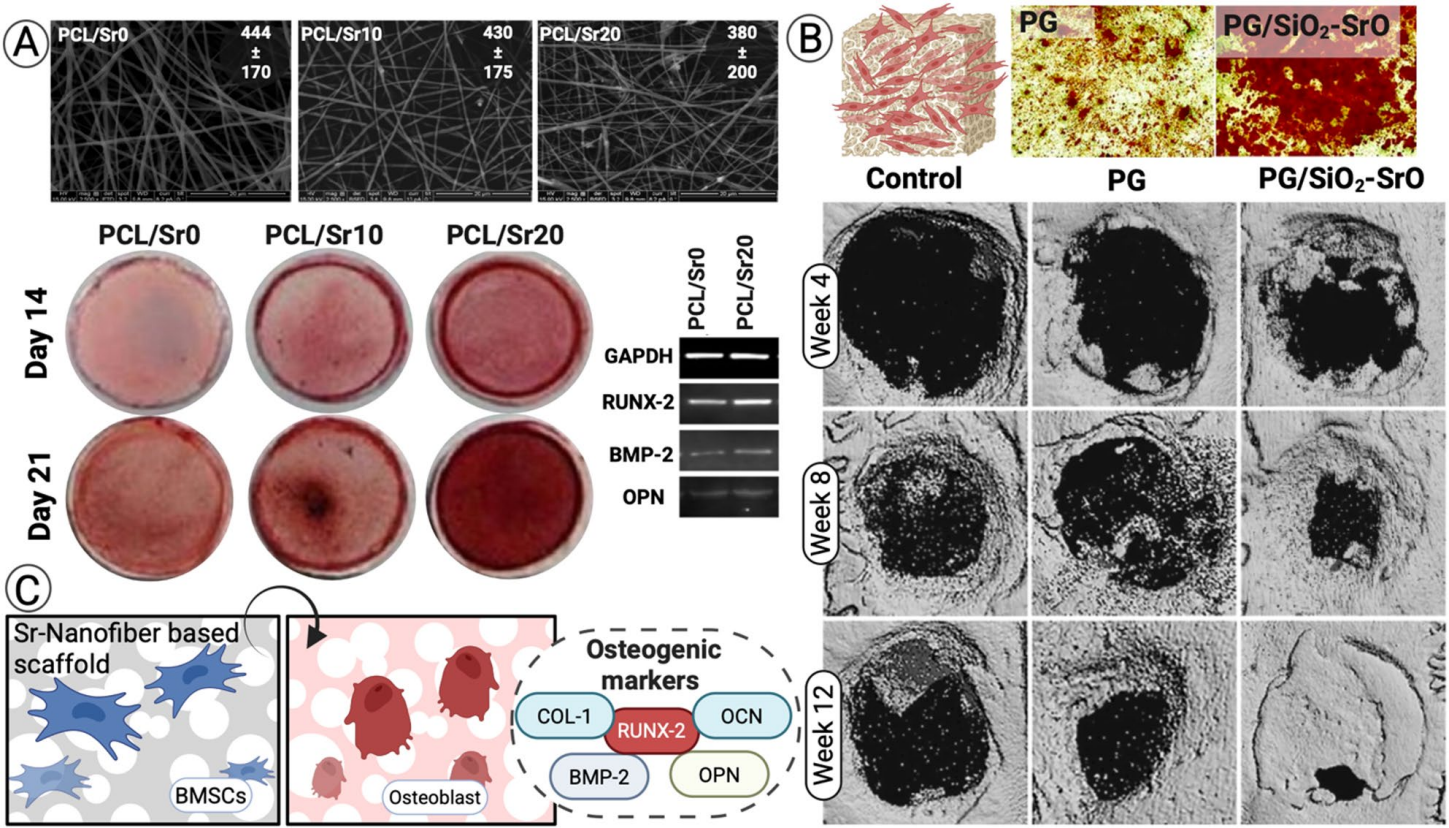
Sr-enhanced osteogenic potential in vitro and in vivo. (**A**) SEM images of PCL/Sr nanofibers at different concentrations (0, 10, and 20%) highlighting changes in fiber diameter. Alizarin Red staining of BMSCs showing Sr-dependent mineral deposition. PCR confirmed upregulation of RUNX-2, BMP-2, and OPN. Adapted with permission from Meka et al. [53]. Copyright Elsevier. (**B**) Alizarin Red staining of BMSCs seeded on poly(lactic acid)/gelatin (PG) scaffolds alone or PG/SiO₂–SrO nanocomposites, showing superior mineralization with Sr-containing scaffolds. In vivo evaluation of calvarial defects treated with the scaffolds revealed enhanced bone regeneration with PG/SiO₂–SrO scaffolds, achieving nearly complete defect healing by day 21 compared to controls and PG alone. Adapted with permission from Cui et al. [162]. Copyright Oxford University Press. (**C)** Schematic of BMSCs osteogenic differentiation on Sr-containing scaffolds. Created in BioRender

Incorporation of Sr into 3D nanofiber scaffolds significantly enhances bone mesenchymal stem cells (BMSCs) proliferation and osteogenic gene expression. Chen et al. showed that Sr-decorated 3D scaffolds (3DS-Sr) upregulated RUNX2, COL-I, OCN, and OPN, and promoted mineralized nodule formation confirmed by alkaline phosphatase (ALP) and alizarin red staining (ARS). In vivo, implantation in rat cranial defects demonstrated superior new bone formation compared to Sr-free scaffolds [31, 161]. Similarly, Xiao et al. demonstrated that mesh-like Sr-HAp nanofiber scaffolds (Mesh@Sr-HAp) promoted BMSC viability, osteogenic gene expression, and bone mineralization, with nearly complete defect closure in OVX rat cranial models (97–99% at 4–8 weeks) [42]. According to meka et al. increasing the concentration of Sr in PCL nanofibers, results in higher osteogenic markers (RUNX-2, BMP-2, and OPN) **(**Fig. 6A**)** [53]. Recently, cui et al. demonstrated enhanced bone regeneration at 12 weeks in vivo calvarial defects model for PG/SiO₂–SrO scaffolds compared to control and PG only **(**Fig. 6B**)** [162].

Human stem cells. In hMSCs, SrF₂-doped nanofibers increased ALP activity, calcium secretion, and ECM mineralization, confirming that Sr²⁺ release supports differentiation without cytotoxicity [48, 163, 164]. Optimal Sr concentrations (10⁻⁵–10⁻⁴ M) stimulate osteoblast proliferation *via* multiple pathways including CaSR, ERK1/2-MAPK, NFATc/Wnt, and PI3K/Akt [165, 166].

#### Osteoblast differentiation

Sr-substituted HAp nanofibers enhanced proliferation of MG63 osteoblast-like cells, ALP activity, and expression of osteogenic markers (RUNX2, COL-I, OCN, BSP), outperforming pure HA scaffolds. The higher solubility of Sr-HAp and its ability to activate ERK1/2 signaling may underlie this effect [167–169]. Composite nanofibers, such as PLA/HA@Sr-HAp/Cu/PPy, further improved osteogenic protein expression by synergizing Sr²⁺ and Cu²⁺ release while preventing nanoparticle aggregation [45] .

#### Inhibition of osteoclastogenesis

**Fig. 7:**
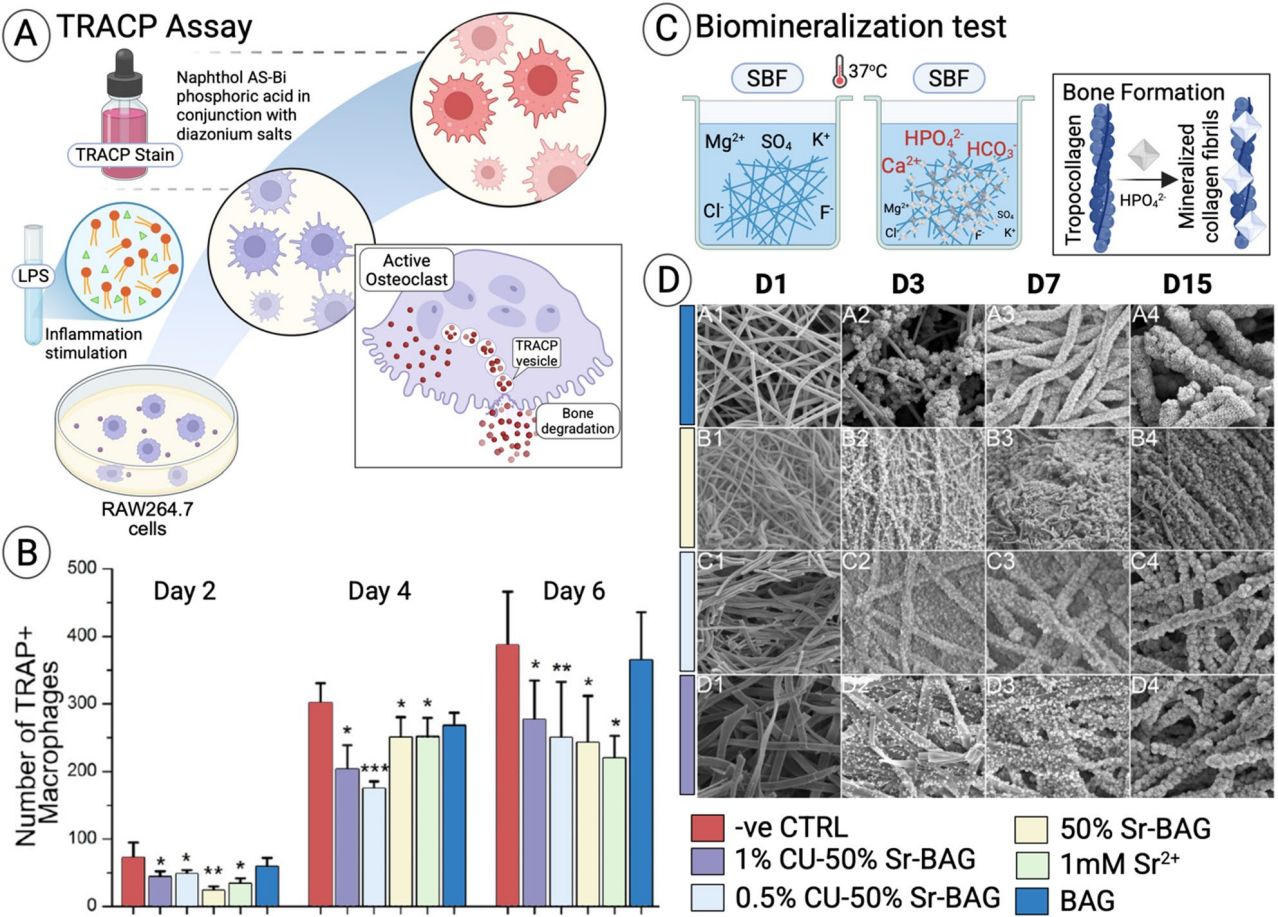
Assessment of Sr-based nanofibers activity. (**A**) Schematic illustration of the TRAP assay concept for detecting activated macrophages, resembling osteoclast activation. (**B**) Bar graph demonstrating that conditioned media from Sr-based nanofibers inhibited osteoclast activity. (**C**) Schematic illustration of the biomineralization assay, showing the apatite-forming ability of nanofibers upon immersion in simulated body fluid (SBF) and the subsequent formation of hydroxyapatite (HPO₄²⁻), mimicking collagen fibril mineralization during bone formation. (**D**) SEM images show different concentrations of Sr-based nanofiber formulations at multiple time points (days 1, 3, 7, and 15). Adapted with permission from Weng et al. [170]. Copyright ACS Applied Materials & Interfaces. Created in BioRender

Beyond stimulating osteoblasts, Sr suppresses osteoclast activity. Mesh@Sr-HAp nanofibers reduced tartrate-resistant acid phosphatase (TRAP)-positive multinuclear cells, disrupted F-actin ring formation, and downregulated osteoclast-related genes such as NFATc1, osteoclast-associated receptor (OSCAR), TRAP, and cathepsin K, in bone marrow mononuclear cells [42]. Weng et al. (2017) demonstrated that electrospun bioactive glass nanofibers doped with Sr^2+^ (substituted for Ca^2+^) and copper release ions for up to four weeks, which not only enhance osteoblast and endothelial cell activity but also suppress the formation of osteoclasts—effectively inhibiting bone-resorbing cells (osteoclasts) while promoting bone formation **(**Fig. 7A and B) [170]. Mechanistically, Sr²⁺ modulates the RANK/RANKL/OPG axis by promoting osteoprotegerin (OPG) binding to RANKL, thereby preventing osteoclast maturation and resorption. Effective inhibition was observed at 0.2–1.5 mmol/L Sr²⁺, particularly with 50% Sr-HAp [166, 171, 172].

#### Mineralization and ECM deposition

Sr-containing nanofibers promoted ECM mineralization in a dose-dependent manner. Increased Sr-Ran incorporation enhanced calcium deposition and collagen mineralization, consistent with Wnt/β-catenin pathway activation [36, 39, 167]. Furthermore, Weng et al. (2017) reported that Sr–Cu co-doped bioactive glass nanofibers demonstrated markedly enhanced apatite-forming ability in simulated body fluid, confirming their superior biomineralization capacity, which in turn supports extracellular matrix deposition and osteogenic differentiation **(**Fig. 7C and D) [170] (Table 2).

**Table 2:** Effects of Sr^2+^ ions in biological systems including anti-inflammatory and Immunomodulatory effects, antioxidant effects, angiogenesis and vascularization support, and antimicrobial properties

Effects	Composite	In vitro cell/ bacteria type	In vitro test	Biological effects	Ref
**Anti-inflammatory and immunomodulatory effects**	Sr-Ran-infused lactoferrin-loaded titanium	RAW264.7	• Immunofluorescence staining. • Semi-quantitative pictures. • The qPCR analysis • The Western blot qualitative.	• High expression of CD206 on STN, TCEP-STN, and LF/TCEP-STNTN surfaces more than TN indicating activation of M2, anti-inflammatory macrophage. • The expression of iNOS was opposite to CD206, indicating low expression of M1 macrophage, pro-inflammatory macrophage. This was due to presence of Sr and LF on the surface.	[140]
	K/HA/Sr-Ran hydrogel	RAW264.7	• Expression of mRNA of inflammatory factor genes; IL-6, IL-1β, and TNF-α	• K/HA/0.5 mM Sr-Ran hydrogel had the most anti-inflammatory effect than others due to the introduction of Sr-Ran into the hydrogel.	[141, 145]
	Sr-Ran on Ti	RAW264.7	• mRNA levels test • Western blot analysis	• Sr^2+^ ions obviously decreased the expression of pro-inflammatory cytokines (TNF-α, IL-1β, and IL-6). • Sr^2+^ ions reduced osteoclastogenesis, inflammatory cell infiltration, and bone loss.	[144]
**Antioxidant effects**	PCL/AsP/Sr-polyP	------	• Radical scavenging activity	• The IC_50_ value, which reveals the antioxidant activity, indicated that 20% was a more efficient antioxidant than 10% ascorbyl palmitate–PCL fiber mats. • The samples exhibited a stable antioxidant capability after 7 days.	[44]
	PVA/SG/Sr-Ber-CQD	------	• DPPH assay	• Sr-Ber-CQD exhibited antioxidant activity that prevented the adverse effects of oxidative stress and impaired wound healing.	[137]
	3DS-E	------	• DPPH assay	• The scaffold showed antioxidant activity, which was enhanced with the increase of SF, due to its tyrosine and tryptophan composition.	[102]
**Angiogenesis and vascularization support**	Mesh@Sr-HAp	HUVECs	• Transwell migration assay. • Tube formation assay • Gene expression analysis	• Enhanced cell migration promotes wound closure. • High level of tube formation rate. • High expression of HIF-1α and VEGF proteins.	[42]
	3DS-Sr	HUVECs	• Immunofluorescence staining • Tube formation assay • Gene expression analysis • Western blot analysis	• Significantly increased mRNA levels of angiogenesis-related genes. • Increased tube-like networks, high numbers of nodes, mesh structures, and elongated branches. • Formation of H-type blood vessels.	[31]
	PLA/HA@Sr-HAp/Cu/PPy	VECs	• CCK-8 test • Cytocompatibility test	• High surface cell activity. • Good biocompatibility.	[45]
	DMSNs/Sr-HAp@PGP	HUVECs	• Expression of angiogenesis-related genes	• High numbers of capillary-like networks. • High expression of angiogenesis genes and proteins.	[103]
	PG/SiO_2_-SrO	HUVECs	• Transwell migration assay • The scratch wound assay • In vitro angiogenesis assay (Tube formation assay)	• PG/SiO_2_-SrO-2 group exhibited the highest cell migration. • PG/SiO_2_-SrO-2 group demonstrated rapid wound closure. • PG/SiO₂-SrO-2 exhibited superior angiogenic potential in tube formation assays.	[162]
	PCL/DFO/Sr-HAp	BMSCs	• Vascular endothelial growth factor (VEGF)	• High VEGF expression due to DFO/Sr-HAp synergistic effect on pro-angiogenesis.	[139]
**Antimicrobial properties**	PCL-SrF_2_	*P. aeruginosa* ATCC 27,853	• Anti-biofilm assay	• Prevent formation of *P. aeruginosa* biofilm.	[48]
	PLA/HA@Sr-HAp/Cu/PPy	*E. coli* and *S. aureus*	• Plate method • Counting method	• There were no colonies. • Showed 100% antibacterial inhibition rate due to the presence of Cu.	[45]
	PVA/Sr-HAp (5 wt%, 10 wt%, and 15% of Sr-HAp)	*E. coli* and *S. aureus*	• Inhibition zone	• PVA/Sr-HAp15 showed an inhibition zone of (15.2 ± 0.2) and (14.5 ± 0.8) against *E. coli* and *S. aureus*, respectively.	[158]

LF/TCEP-STN, lactoferrin loaded into strontium-doped nanotubes modified by tris-(2-carboxyethyl)-phosphine.; K/HA/Sr-Ran hydrogel, strontium ranelate into the keratin/hyaluronic acid hydrogel; Sr-Ran on Ti, strontium ranelate on the titanium surface; PCL/AsP/Sr-polyP, ascorbyl palmitate–polycaprolactone fiber mats loaded with strontium polyphosphate nanoparticles; PVA/SG/Sr-Ber-CQD, strontium-doped berberine carbon quantum dots incorporated into polyvinyl alcohol and scleroglucan-based nanofiber; 3DS-E, 3D scaffold modified with metal phenolic networks composed of epigallocatechin gallate and Sr^2+^ ions; Mesh@Sr-HAp, strontium-hydroxyapatite-enriched polycaprolactone/silk fibroin nanofibers; 3DS-Sr, 3D nanofiber scaffolds decorated with strontium nanoparticles; PLA/HA@Sr-HAp/Cu/PPy, polylactic acid/hydroxyapatite nanofiber coated with strontium-doped hydroxyapatite/copper/polypyrrole composite; DMSNs/Sr-HAp@PGP, short nanofibers containing dimethyloxalylglycine-loaded mesoporous silica nanoparticles with a 3D printed strontium-contained hydroxyapatite/polycaprolactone scaffold; PG/SiO_2_-SrO, poly (lactic acid)/gelatin and silica-strontium oxide electrospun short fibers; PCL/DFO/Sr-HAp, desferrioxamine and strontium-doped hydroxyapatite loaded into core and shell of PCL-based nanofiber membrane; PCL-SrF_2_, strontium fluoride incorporated into PCL nanofiber membrane; PVA/Sr-HAp, strontium-substituted hydroxyapatite incorporated into electrospun polyvinyl alcohol nanofiber scaffolds; P. aeruginosa, *Pseudomonas aeruginosa;* E. coli, *Escherichia coli;* S. aureus, *Staphylococcus aureus*

## Biomedical applications for Sr-Based nanofibers

**Fig. 8:**
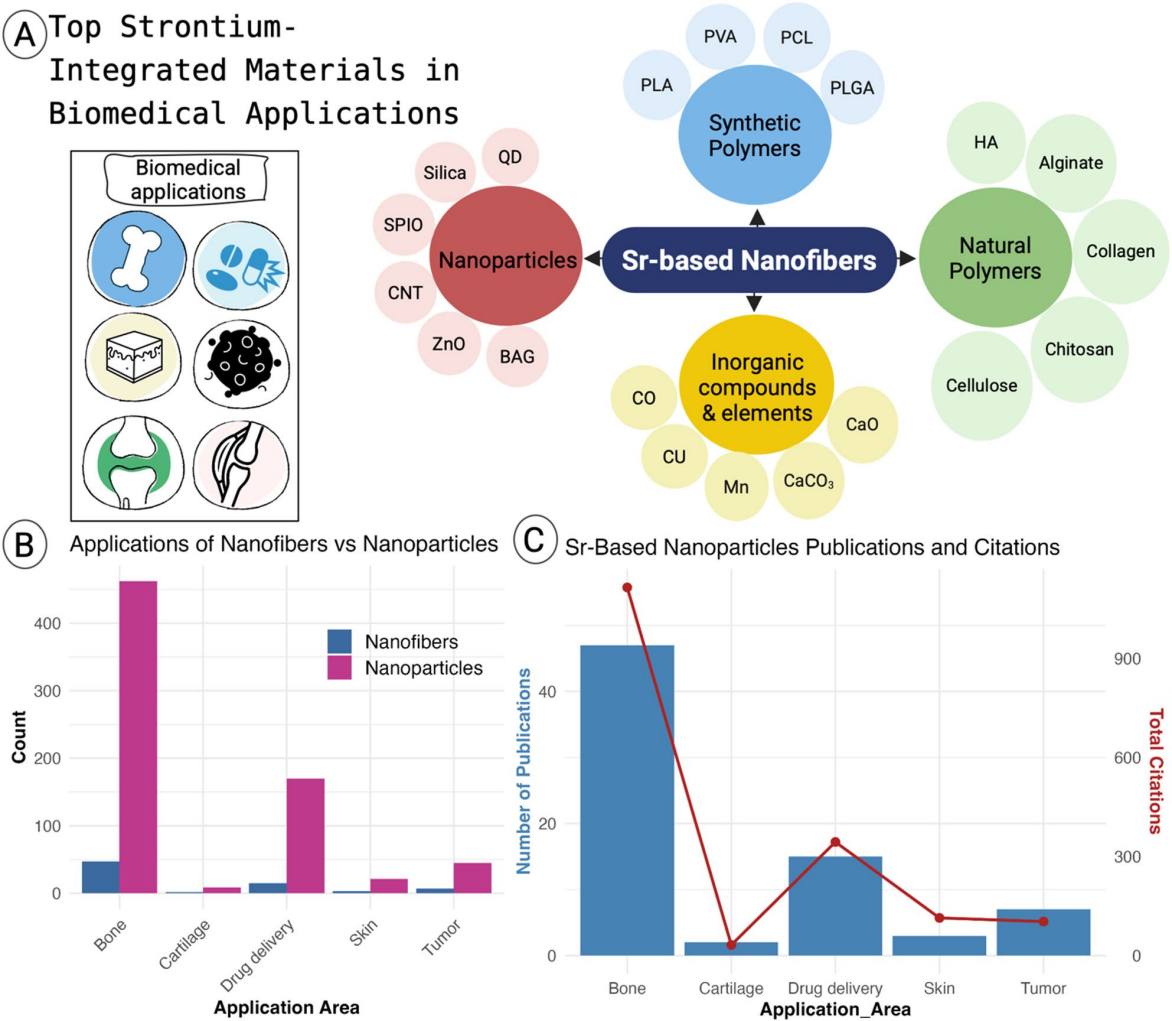
Applications and publication trends of Sr-based nanofibers. (**A**) Schematic of major biomedical applications. (**B**) Comparison of publications on Sr-based nanofibers and nanoparticles. (**C**) Publication and citation trends across biomedical applications (Web of Science, Jan 2026)

Bibliometric analysis of publications reveals that Sr has been widely integrated with various polymers and nanoparticles to generate multifunctional nanofibers for biomedical applications. These combinations include both synthetic and natural polymers, as well as diverse nanoparticle systems. Among biomedical applications, bone tissue engineering has received the most attention, with 44 publications and nearly 1,000 citations. The second most common application of Sr-based nanofibers is in drug delivery, followed by studies related to tumor therapy. A smaller number of studies have explored their use in skin repair and cartilage regeneration. While Sr-based nanoparticles have been tested in tendon repair, no studies to date have investigated Sr-based nanofibers for tendon regeneration **(**Fig. 8**)**.

### Sr-based nanofibers in bone regeneration

Sr is one of the trace elements in the human body that are obtained after digestion and stored in bone and teeth. Sr^2+^ ions gains its biological activity from its similarity to Ca^2+^ ions in size and charge. Sr^2+^ ions have been reported to promote osteogenic differentiation of BMSCs *via* its dual role during cell metabolism, where induce osteoblastic differentiation from human MSCs (hMSCs), increasing osteoblast proliferation and decrease osteoblast apoptosis, and decrease osteoclast differentiation leading to osteoclast apoptosis [173]. Additionally, Sr has the ability to increase bone mass, density, and mechanical properties while reduce the risk of bone fractures [174]. However, long-term and high dose of Sr intake has side effects and can cause high rickets [175].

Clinically, Sr-based interventions have been extensively investigated for bone-related disorders. A search of the ClinicalTrials.gov database indicates that Sr-based interventions have been featured in approximately 46 clinical studies, primarily focused on fracture prevention and cancer-related bone pain. Among these, at least 31 trials have been marked as completed, supporting a substantial evidence base for Sr’s clinical investigation. For instance, several trials have evaluated Sr-Ran in postmenopausal osteoporosis including the landmark Spinal Osteoporosis Therapeutic Intervention (SOTI) and Treatment of Peripheral Osteoporosis (TROPOS) phase III studies demonstrating significant reductions in vertebral (≈ 41%) and hip (≈ 36%) fractures [176]. Other completed trials have investigated Sr’s pharmacokinetics (e.g., with Sr lactate), safety in bone healing post-surgery, its use in knee osteoarthritis, and combinations with other treatments [177, 178].

In this regard, incorporation of Sr into nanofiber scaffold is the perfect choice for sustain release of Sr^2+^ ions and long duration effect without causing any toxicity to the cells. So that, during osteogenesis, Sr can regulate and affect various types of cells such as MSCs, osteoblast, and osteoclast to promote bone repair and regeneration.

Among the materials employed, HAp was identified as the top material integrated with Sr-based nanofibers in bone regeneration studies. This reinforces our earlier observation that HAp nanoparticles dominate the literature as the most extensively investigated material for promoting osteoblast activity [179]. Sr-based nanofibers have demonstrated strong potential in bone tissue engineering by enhancing stem cell proliferation, osteogenic differentiation, mineralization, and suppression of osteoclast activity as summarized in Table 3.

### Sr-based nanofibers in articular cartilage regeneration

**Fig. 9:**
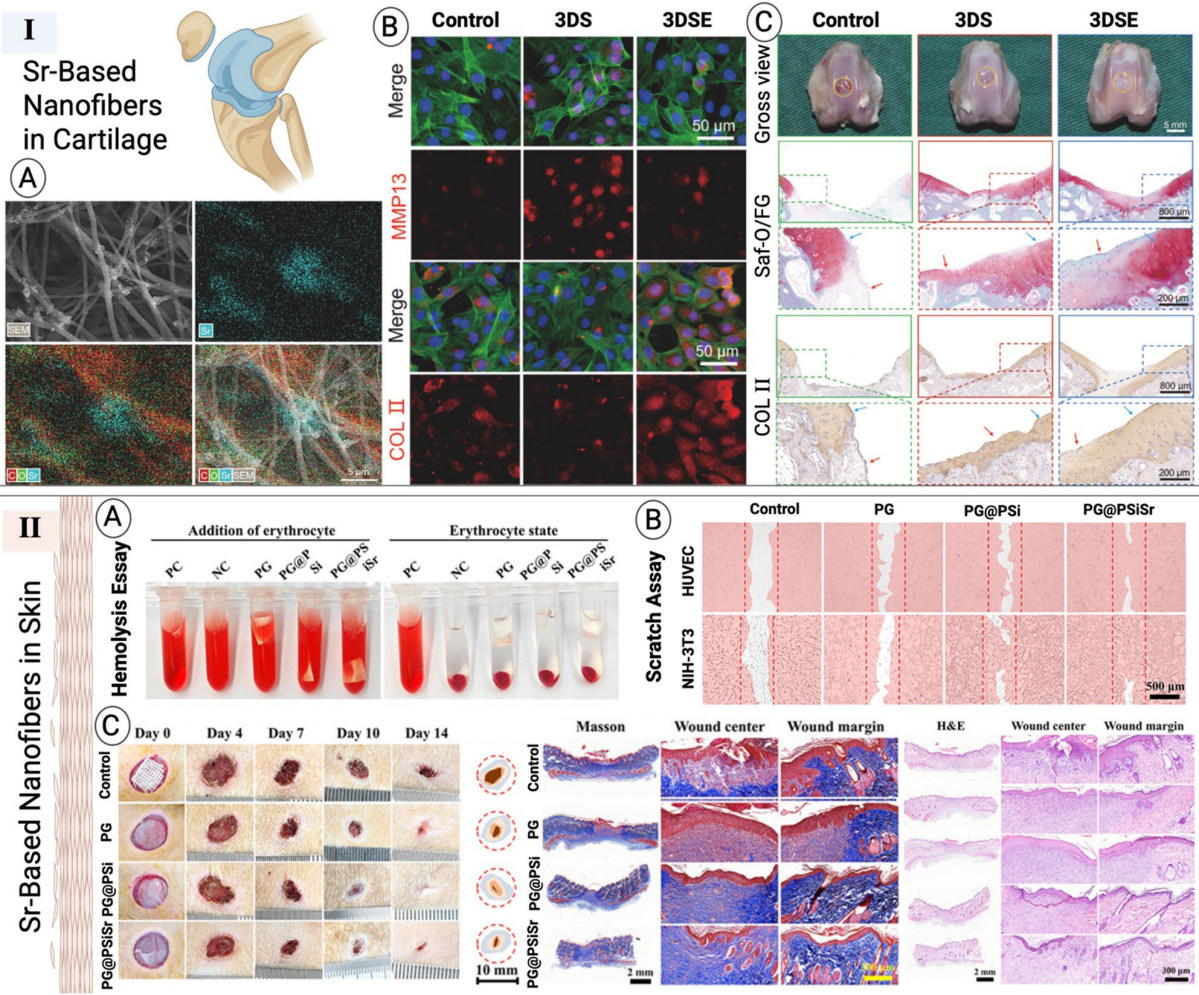
Biomedical applications of Sr-based nanofibers. **I. Cartilage: **(**A**) Elemental mapping of the 3DS-E scaffold (3DS-E: Sr/EGCG-modified 3D PLCL/SF scaffold). (**B**) In vitro chondroprotective marker expression. (**C**) In vivo rabbit model: gross morphology, H&E staining, Safranin-O/Fast Green, and COL II immunohistochemical staining of repaired cartilage. Copyright obtained from Chen et al. [102] (Elsevier). **II. Skin**: (Mesoporous silicon with Sr-powered PLGA/gelatin-based dressings). (**A**) Hemolysis assay. (**B**) Scratch assay. (**C**) In vivo rat excisional wound model showing wound healing of treatment groups, gross morphology, Masson’s trichrome staining, and H&E staining. Copyright obtained from Li et al. [182] (Taylor & Francis)

Acute inflammation following cartilage injury disrupts the healing cascade, as the persistent release of pro-inflammatory cytokines suppresses cartilage matrix synthesis [155]. Sr has shown promise in addressing this challenge, given its dual capacity to promote cartilage matrix remodeling and stimulate chondrogenic differentiation of stem cells [180], while simultaneously attenuating inflammation. In particular, Sr has been reported to modulate macrophage phenotype, thereby reducing the expression of pro-inflammatory cytokines and fostering a more regenerative environment [155, 180]. For example, Chen et al. demonstrated that a three-dimensional scaffold modified with metal–phenolic networks composed of epigallocatechin gallate and Sr²⁺ (3DS-E) effectively downregulated inflammatory gene expression in chondrocytes, enhanced matrix secretion, and promoted cartilage regeneration **(**Table 3**)** [102]. Moreover, 3DS-E protected chondrocytes under inflammatory stress by activating the nuclear factor erythroid 2-reladed factor 2 (Nrf2) pathway, thereby preventing matrix degradation and supporting cartilage remodeling. Complementary findings were reported by Fenbo et al. who developed a strontium chondroitin sulfate/silk fibroin (Sr-CS/SF) membrane with a microporous structure [169]. Their work highlighted the ability of Sr-CS/SF membranes to not only improve mechanical and physicochemical properties but also to modulate macrophage responses, reducing catabolic gene expression while enhancing osteogenic signaling. Although conducted in the context of guided bone regeneration, these results reinforce the broader concept that Sr^2+^ ions can orchestrate immunomodulatory and matrix-preserving effects, underscoring their translational potential in cartilage tissue engineering (Fig. 9).

### Sr-based nanofibers in skin regeneration

The concept of incorporating Sr into wound dressings was first proposed in 2020 by Dodero et al. who developed alginate-based electrospun membranes crosslinked with Sr^2+^ ions. Their work focused primarily on physicochemical and biological characterization, reporting favorable cell adhesion, mechanical strength, and water vapor permeability, suggesting that Sr-crosslinked mats could be promising wound dressing candidates (Table 3) [108]. Building on this, Pálos et al. (2024) further investigated Sr-containing polysuccinimide (PSI)-based scaffolds, emphasizing cytotoxicity, antibacterial activity, and mechanical performance [181]. More advanced Sr-based nanofibrous systems have incorporated strontium-doped mesoporous silicon (PSiSr) into electrospun PLGA/gelatin fibers, enabling sustained Sr²⁺ and Si⁴⁺ release and significantly enhancing angiogenesis, cell migration, collagen deposition, and wound closure in full-thickness rat skin defects, thereby reinforcing the translational potential of Sr-enriched nanofibrous dressings for skin regeneration [182]. They demonstrated that Sr(NO_3_)_2_ was safely incorporated into the nanofibrous system, with complete release within 8 h, and reported no cytotoxicity toward healthy or tumor cells, further reinforcing the potential utility of Sr for wound-related applications. Most recently, this concept has been translated into preclinical evaluation, as demonstrated by Karimi et al. (2025), who fabricated PVA/SG/Sr-Ber-CQD nanofibrous dressings and validated their efficacy in full-thickness skin wounds [137]. Importantly, the Sr-enriched dressing achieved wound healing outcomes comparable to a commercial product, thus providing the first in vivo evidence supporting the translational promise of Sr-based nanofibers for skin regeneration.

### Sr-based nanofibers in drug delivery and tumor therapy

**Fig. 10:**
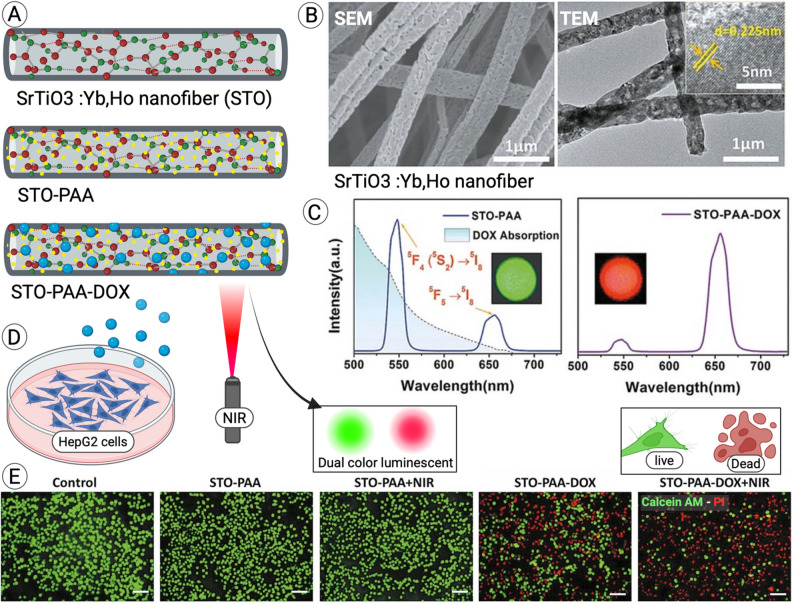
(**A**) Schematic illustration of SrTiO₃:Yb, Ho (STO) nanofibers functionalized with polyacrylic acid (PAA) and loaded with doxorubicin (DOX), Created in BioRender. (**B**) SEM and TEM images of STO nanofibers. (**C**) Upconversion emission spectrum and fluorescence image (inset) of STO-PAA nanofibers; UV/Vis absorption of DOX and upconversion spectrum with fluorescence image (inset) of STO-PAA-DOX. (**D**) Schematic showing NIR-triggered dual-color luminescence and DOX release tested in vitro on HepG2 cells. (**E**) Live/dead staining (Calcein-AM: green; PI: red) of HepG2 cells cultured with different materials, showing the highest cytotoxicity in the STO-PAA-DOX + NIR group. Adapted with permission from Fu et al. [184]. Copyright Wiley

Recent advances have expanded the application of Sr-based nanofibers beyond their classical role in osteogenesis toward multifunctional drug delivery platforms. Doping rare earth ions to SrTiO₃ nanofibers have been engineered to integrate both therapeutic release and optical monitoring. For example, pH-triggered erbium-doped SrTiO3 (SrTiO₃:Er) nanofibers enable controlled drug delivery in response to the acidic microenvironment commonly associated with inflamed or tumor tissues, while simultaneously providing optical feedback to monitor release dynamics (Table 3) [183]. Similarly, SrTiO₃:Yb, Ho (STO-PAA) nanofibers have been constructed for near-infrared (NIR)-triggered chemotherapy, where localized light exposure not only induces doxorubicin (DOX) release but also permits dual-color luminescent imaging, thereby coupling therapy with real-time monitoring (Fig. 10) [184]. These systems highlight the versatility of Sr nanofibers as implantable, responsive carriers capable of site-specific, externally controlled drug delivery, while simultaneously offering a non-invasive means of tracking therapeutic outcomes [185]. Such designs position Sr nanofibers at the interface of regenerative medicine, cancer therapy, and precision drug delivery, underscoring their potential to address both efficacy and safety challenges in next-generation biomedical applications.

In parallel, Sr has demonstrated promising utility in oncology, particularly in bone metastasis management, as evidenced by multiple clinical studies. Notably, a randomized Phase III trial (NCT00365105) evaluated the addition of Sr-89 to standard therapy (zoledronate, vitamin D, and calcium) in patients with prostate, lung, or breast cancer bone metastases, exploring its role in preventing or delaying skeletal complications [186]. Earlier Phase II data also illustrated meaningful pain palliation in prostate cancer patients using Sr-89, with sustained decreases in pain intensity and frequency and favorable tolerability compared to chemotherapy [187]. More recently, a Phase IV study (NCT05466812) is investigating Sr-89 in differentiated thyroid cancer with bone metastases, extending its potential beyond symptom relief to include tumor-specific biomarkers and imaging outcomes [188].

Building on these findings, Sr-based nanofibers offer a unique platform for localized cancer therapy and bone regeneration. Two translational pathways have been proposed: (1) local bone-repair scaffolds for tumor-induced or metastatic bone defects, leveraging Sr’s osteogenic, antibacterial, and immunomodulatory effects, and (2) multifunctional drug-delivery nanofibers that combine local cytotoxicity with osteo-supportive benefits. For instance, electrospun Sr-doped bioglass and HAp nanofibers have demonstrated enhanced bone regeneration and immune modulation, supporting their use as scaffolds for restoring bone integrity after oncologic resection [38, 189]. Sr-Ran–containing nanofibrous scaffolds exhibited cytocompatibility with bone-marrow stromal cells and osteo-supportive characteristics, suggesting potential utility in repairing tumor-associated bone defects [80]; however, no in vitro or in vivo anti-tumor/metastasis experiments were performed, so anti-metastatic effects remain hypothetical pending direct evaluation. Second, multifunctional delivery platforms based on Sr-eluting nanofibers allow controlled nanofibers promote stem-cell osteogenesis [53], and more recent studies explored Sr-decorated nano-systems as carriers for anticancer drugs, thereby combining local cytotoxic effects with osteo-supportive benefits [190]. Another study demonstrated that the presence of different concentration (5, 7.5, and 10%) of Sr-nitrate/PSI nanofibers (PSI/ Sr(NO_3_)_2_ electrospun membrane) have no cytotoxic effect against MG-63 tumor cell or 155BR fibroblasts healthy cells [181]. According to zare et al. incorporating Sr-bioactive glass nanofibers (5%, 10%, and 15% w/w) into sodium alginate (SA) hydrogel scaffolds enhanced the viability of MG-63 cells with the 15% w/w of Sr-BG contents [38].

**Table 3:** Biomedical applications of Sr-based nanofibers including bone regeneration, articular cartilage regeneration, drug delivery and tumor treatment, and skin regeneration

Application	Composite	In vitro cell	In vitro and/or in vivo test name	In vitro activity	In vivo model and activity	Ref
**Bone regeneration**	PVA-Sr-Ran/PCL	MSCs	• ALP activity • RT-PCR • ARS activity	• High expression of osteogenic-related genes	• Sr-Ran: Improved bone formation of calvarial defects in a Wister rat model	[39]
	Sr-HA/PVP	MG63 like-osteoblast	• ALP assay • qPCR analysis	• Higher proliferation rate (2.39) • ALP activity increased, which enhanced mineralization • High expression of osteogenic-related genes	--	[167]
	PLA/HA@Sr-HAp/Cu/PPy	Osteoblast (From the skull of an SD rat)	• ALP activity • ARS staining • qRT-PCR • Western blot analysis	• High expression of osteogenesis-related genes (COL-I, Runx-2, OCN, HIF-1α and S100A10) and proteins • High proliferation rate • Positive effect for bone formation in early and late stages	--	[45]
	PCL-SrF_2_	hMSCs	• ALP assay • Arsenzo III assay	• High ALP activity and biomineralization increased • Cell adhesion, attachment, and proliferation increased	--	[48]
	DMSNs/Sr-HAp@PGP	BMSCs	• Micro-CT analysis • ALP activity • ARS activity • qPCR analysis • Western blot	• High expression of osteogenic-related genes • Well mineralization • High calcium deposition enhanced matrix mineralized formation	• New bone formation in BLABc mouse model.	[103]
	3DS-Sr	BMSC	• Immunofluorescence staining • qPCR analysis • Western blot analysis • ALP activity • ARS activity	• High expression of osteogenic genes (COL-I, OPN, RUNX2, OCN, FZD8, and β-catenin) • High mineralization	• Formation of new bone in SD rats. • Increased cell proliferation.	[31]
	Mesh@Sr-HAp	BMSCs	• ALP assay • ARS assay • Osteogenesis-related gene expression	• High cell density • High ALP and ARS activity leading to high mineralization	• High bone defect closure rate in SD rats.	[42]
		BMMC	• TRAP staining • Fusion rates and actin ring formation analysis. • qRT-PCR assay	• Reduced number of TRAP-positive cells • Reduced F-actin ring area • Suppression of osteoclastic markers NFATc1, OSCAR, TRAP, and cathepsin K	--	
	TPU/Sr-HAp (TS-1, TS-3, TS-5 & TS-7)	gMSCs	• MTT assay • ALP assay • ALP level • ARS staining • In vitro biomineralization.	• The Sr-HAp facilitated cell adhesion and growth • TS-3 scaffolds showed maximum cell viability • TS-5 scaffolds showed high level of ALP, OCN and RUNX2 • TS-5 scaffolds demonstrated biomineralization	--	[136]
	PG/SiO_2_-SrO (SrO-1, SrO-2, SrO-3) PG: (PLA/Gel aerogel scaffolds)	BMSCs	• ALP assay • ARS staining • RT-qPCR	• PG/SiO_2_-SrO showed the strongest induction of ALP activity • PG/SiO2-SrO-2 group displayed the highest number of Ca nodules and mineralization • High expression of COL-1, OCN, RUNX-2 and OPN genes.	• The PG/SiO_2_-SrO scaffold group demonstrated the highest new bone coverage in week (12) in rat calvarial defect model.	[162]
	PCL/DFO/Sr-HAp	BMSCs	• ALP staining and activity • ARS activity • RT-PCR	• ALP activity was the highest for S6 • The S6 group showed the best ability to form a mineralized calcium nodule. • High level of HIF-α. • High value of BMP-2, OPN and OCN genes.	--	[139]
**Articular cartilage regeneration**	Sr-CS/SF	RAW 264.7	• Cell culture. • Cell proliferation assay of osteoblasts. • Real-time PCR analysis	• Modulate macrophage responses. • Reducing catabolic gene expression. • Enhancing osteogenic signaling.	--	[169]
	3DS-E	chondrocyte	-----	• Suppress expression of inflammatory-related genes. • Activate the Nrf2 pathway, which protects chondrocytes from inflammation.	• Formation of cartilage-like tissues in rabbits. • High ECM accumulation and COL II, which enhance the formation of natural cartilage.	[102]
**Skin regeneration**	SA/PEO@Sr	Mouse fibroblast L929 cell line	• Wound healing patches • Cytotoxicity assay • Cell adhesion and viability tests • Protein adsorption test	• SA/PEO membrane crosslinked with Sr and Ba are biocompatible. • Cell adhesion and growth were perfect.	--	[108]
	PVA/SG/Sr-Ber-CQD	Human dermal fibroblasts (HDF)	• Cell migration assay • Cell viability analysis	• All nanofiber mats do not exceed available value (< 2%), exhibiting no hemolysis. • 5% Sr-Ber-CQD had the biggest impact on all the groups in migration test, repairing 67.77% of the scratches. • Sr-Ber-CQD increased cell proliferation, down-regulated matrix metalloproteinase (MMP9), and up-regulated transforming growth factor-β1 (TGF-β1), leading to accelerated wound healing.	• The nanofibers containing 5% Ber.Sr-CQD exhibit more efficient wound healing compared to NF/0%Ber.Sr-CQD in Wister rats.	[137]
**Drug delivery and tumor treatment**	SrTiO₃:Er	-	• PH-study at physiological and acidic conditions. • Optically monitored drug delivery near-infrared spectrum (∼980 nm).	• The drug loading capacity of SrTiO₃:Er nanofibers is increased when amino group was functionalized on the surface. • The rug release was enhanced at acidic pH of 4.7.	• --	[183]
	STO-PAA-DOX (STO = SrTiO_3_:Yb, Ho)	Hep-G2 (human liver cancer cells)	• Cell viability	• STO nanofibers have a slight effect on Hep-G2 cells. • Inducing NIR, the effect is enhanced and kills the cells. • NIR in STO-PAA-DOX nanofiber induced cells killing but with stronger effect.	• --	[184]
	SrTiO₃:Er/DOX	Hep-G2 cells	• Cell viability • Drug release	• SrTiO₃:Er showed highest DOX loading capacity and sustained releasing kinetics. • SrTiO₃:Er/DOX demonstrated stronger in vitro anticancer efficacy against Hep-G2 cells. • SrTiO₃:Er nanofibers have ratiometric-monitored DOX release functionalities.	• --	[185]

PVA-Sr-Ran/PCL, strontium ranelate/polyvinyl alcohol and polycaprolactone-based nanofibers; Sr-HAp/PVP, strontium-hydroxyapatite loaded into poly(vinylpyrrolidone) nanofibers; PLA/HA@Sr-HAp/Cu/PPy, polylactic acid/hydroxyapatite nanofiber coated with strontium-doped hydroxyapatite/copper/polypyrrole composite; PCL-SrF_2_, strontium fluoride incorporated into PCL nanofiber; DMSNs/Sr-HAp@PGP, short nanofibers containing dimethyloxalylglycine-loaded mesoporous silica nanoparticles with a 3D printed strontium-contained hydroxyapatite/polycaprolactone scaffold; 3DS-Sr, 3D nanofiber scaffolds decorated with strontium nanoparticles; Mesh@Sr-HAp, strontium-hydroxyapatite-enriched polycaprolactone/silk fibroin nanofibers; TPU/Sr-HAP, thermoplastic polyurethane elastomer dispersed with strontium-hydroxyapatite nanorods; PG/SiO_2_-SrO, poly (lactic acid)/gelatin and silica-strontium oxide electrospun short fibers; PCL/DFO/Sr-HAp, desferrioxamine and strontium hydroxyapatite loaded into core and shell of PCl-based nanofiber; Sr-CS/SF, strontium chondroitin sulfate/silk fibroin nanofibers; 3DS-E, 3D scaffold modified with metal phenolic networks composed of epigallocatechin gallate and Sr^2+^ ions; SA/PEO@Sr, sodium alginate and poly(ethylene oxide)-based nanofiber crosslinked with strontium; PVA/SG/Sr-Ber-CQD, strontium-doped berberine carbon quantum dots incorporated into polyvinyl alcohol and scleroglucan-based nanofiber; SrTiO₃:Er, Erbium-doped strontium titanate; STO-PAA-DOX, Ytterbium (Yb³⁺) and Holmium (Ho³⁺) co-doped strontium titanate fiber decorated on the surface with polyacrylic acid loaded with doxorubicin; SrTiO₃:Er^3+^/DOX, Erbium-doped electrospun strontium titanate nanofibers loaded with doxorubicin

## Conclusion

Electrospinning could control fiber diameter, surface roughness, porosity, and composite chemical composition that enhance both mechanical properties and bioactivity for specific biomedical applications. Polymeric and ceramic composite, especially Sr-doped hydroxyapatite or bioactive glass proves it excellent biocompatibility as well as control the bioactive Sr^2+^ release. Sr-based nanofibers represent a promising class of multifunctional biomaterials with broad biomedical potential. Several studies have demonstrated that incorporation of Sr^2+^ ions into polymeric or ceramic nanofibers significantly enhances stem cell proliferation, osteogenic differentiation, mineralization, while concurrently suppresses osteoclast activity and inflammatory pathways, thereby establishing a pro-regenerative microenvironment. Additionally, Sr^2+^ ions could not only improve the viability of fibroblasts, endothelial cells, and smooth muscle cells which play a key role in blood vessel formation and angiogenesis but also effectively suppress bacterial growth. Beyond musculoskeletal applications, Sr-incorporated nanofibers have been engineered as advanced drug delivery and theranostic systems, demonstrating efficacy in cancer therapy, wound healing, and other therapeutic contexts. Collectively, these attributes position Sr-based nanofibers as strong candidates for next-generation biomedical scaffolds in tissue engineering and regenerative medicine.

### Challenges and future perspectives

Despite these promising effects of Sr-based nanofibers, some challenges remain such as the fabrication of reproducible large-scale for commercialization, controlling the release kinetics of Sr^2+^ ions or bioactive drugs, limited regenerated capacity of cardiac tissue, no study reported in tendon repair, and long-term in vivo assessment. Future research should focus on developing advanced fabricating technique with specific design and optimizing parameters to fabricate Sr-based nanofibers with controlled morphology for multifunctional purpose, improving the release of bioactive drugs, gene, etc., in sustain long-term period to reach optimal therapeutic concentrations while minimizing the cytotoxicity, elucidating long-term biocompatibility and degradation profiles, and advancing translational studies to bridge preclinical findings with clinical implementation. Since Sr^2+^ ions play role in blood vessel formation, it is recommended for research to focus on the application of Sr-based nanofibers in the developments of vascular graft, tendon repair and cardiac tissue.

## Data Availability

No datasets were generated or analysed during the current study.

## Abbreviations

A-HSBS: Air-heated solution blow spinning
ALP: Alkaline phosphatase
ARS: Alizarin red staining
AsP: Ascorbyl palmitate
AZrO_3_: Alkaline earth zirconates (A= Ca^2+^ and Sr^2+^)
Ba^2+^: Barium ions
bFGF: Basic fibroblast growth factor
BMP-2: Bone morphogenetic protein-2
BMSCs: Bone mesenchymal stem cells
Ca^2+^: Calcium ions
CaSR: Calcium-sensing receptor
CNF: Carbon nanofibers
Co^2+^: Cobalt
COL-I: Collagen type I
Cu: Copper
DOX: Doxorubicin
ECM: Extracellular matrix
ERK: Extracellular signal-regulated kinase
ERK1/2-MAPK: Extracellular signal-regulated kinase-1 / 2-Mitogen-activated protein kinase
F-CNF: Functionalized carbon nanofibers
Fe(NO_3_)_3_: Ferric nitrate
GCE: Glassy carbon electrode
GEL: Gelatin
HAp: Hydroxyapatite
HIF-1α: Hypoxia-inducible factor-1 alpha
HUVEC: Human umbilical vein endothelial cells
ICIE16-BG/Sr: Strontium doped-ICIE16 bioactive glass
IL-1β: Interleukin-1 beta IL-1β
IL-6: Interleukin-6
La_1-x_Sr_x_CoO_3_: Strontium doped-lanthanum cobaltite
Mg^2+^: Magnesium ions
MSCs: Mesenchymal stem cell
MyD88: Myeloid Differentiation primary response 88
NaBH_4_: Sodium borohydride
NFATc/Wnt: Nuclear factor of activated T-cells, cytoplasmic/Wnt signaling pathway
NF-κB: Nuclear Factor kappa-light chain enhancer of activated B cells
Nrf2: Nuclear factor erythroid 2–related factor-2
OA: Osteoarthritis
OCN: Osteocalcin
OPG: Osteoprotegerin
OPN: Osteopontin
OSCAR: Osteoclast-associated receptor
PC: Polycarbonate
PCL: Polycaprolactone
PI3K/Akt: Phosphoinositide 3-kinase/Protein kinase B pathway
PLA: Polylactic acid
PLGA: Poly(lactic-co-glycolic acid)
PPy: Polypyrrole
PSI/Sr(NO_3_)_2_: Strontium nitrate loaded polysuccinimide
PVA: Polyvinyl alcohol
PVP: Polyvinyl pyrrolidone
RANK: Receptor activator of nuclear factor κB
RANKL: Receptor activator of nuclear factor κB ligand
ROS: Reactive oxygen species
RUNX2: Runt-related transcription factor-2
SA: Sodium alginate
SBS: Solution blow spinning
SF: Silk fibroin
SnO_2_: Strontium-doped tin oxide
Sr(NO_3_)_2_: Strontium nitrate
Sr(OH)_2_: Strontium hydroxide
Sr^2+^: Strontium ions
Sr_2_P_2_O_7_@F-CNF: Strontium phosphate functionalized carbon nanofiber composite
Sr_6_Rh_5_O_15_: Strontium rhodium oxide
Sr-Al NPs: Strontium aluminate nanoparticles
Sr-BG: Strontium bioactive glass
Sr-CS/SF: Strontium chondroitin sulfate/silk fibroin
SrF_2_: Strontium fluoride
SrFe_12_O_19_: Strontium hexaferrite
Sr-HAp: Strontium hydroxyapatite
Sr: Strontium oxide
Sr-polyP: Strontium polyphosphate
Sr-Ran: Strontium ranelate
SrTiO_3_: Strontium titanate
SrTiO₃: Er: Erbium-doped strontium titanate
SrZrO_3_/F-CNF: Strontium zirconate functionalized carbon nanofiber
STO-PAA: Ytterbium and holmium co-doped SrTiO₃ decorated on polyacrylic acid surface
Ti(OH)_6_^2-^: Titanium hydroxide
TiO_2_: Titanium dioxide
TLR4: Toll-like receptor-4
TNF-α: Tumor Necrosis Factor-α
TRAP: Tartrate-resistant acid phosphatase
VEGF: Vascular endothelial growth factor
Zn^2+^: Zinc ions
ZnO NPs: Zinc oxide nanoparticles
